# The necroptotic cell death pathway operates in megakaryocytes, but not in platelet synthesis

**DOI:** 10.1038/s41419-021-03418-z

**Published:** 2021-01-28

**Authors:** Diane Moujalled, Pradnya Gangatirkar, Maria Kauppi, Jason Corbin, Marion Lebois, James M. Murphy, Najoua Lalaoui, Joanne M. Hildebrand, John Silke, Warren S. Alexander, Emma C. Josefsson

**Affiliations:** 1 The Walter and Eliza Hall Institute of Medical Research, Melbourne, VIC Australia; 2 Department of Medical Biology, University of Melbourne, Melbourne, VIC Australia

**Keywords:** Necroptosis, Experimental models of disease

## Abstract

Necroptosis is a pro-inflammatory cell death program executed by the terminal effector, mixed lineage kinase domain-like (MLKL). Previous studies suggested a role for the necroptotic machinery in platelets, where loss of MLKL or its upstream regulator, RIPK3 kinase, impacted thrombosis and haemostasis. However, it remains unknown whether necroptosis operates within megakaryocytes, the progenitors of platelets, and whether necroptotic cell death might contribute to or diminish platelet production. Here, we demonstrate that megakaryocytes possess a functional necroptosis signalling cascade. Necroptosis activation leads to phosphorylation of MLKL, loss of viability and cell swelling. Analyses at steady state and post antibody-mediated thrombocytopenia revealed that platelet production was normal in the absence of MLKL, however, platelet activation and haemostasis were impaired with prolonged tail re-bleeding times. We conclude that MLKL plays a role in regulating platelet function and haemostasis and that necroptosis signalling in megakaryocytes is dispensable for platelet production.

## Introduction

Blood platelets are produced from megakaryocytes, large polyploid cells residing in the bone marrow (BM), spleen^1^, and lungs^2^. Megakaryocytes are thought to produce platelets via a cytoskeletal-driven process, in which cytoplasmic protrusions called proplatelets extend from the BM and into the blood stream^3,4^. However, this model for platelet synthesis was recently challenged, because megakaryocyte membrane budding, rather than proplatelet formation, was reported to supply the majority of the platelet biomass^5^. Alternative mechanisms, such as megakaryocyte rupture, have been proposed in response to acute needs^6,7^.

Platelets are small anucleate cells, classically known for their role in haemostasis and thrombosis, staying in circulation up to 10 days in humans and 5 days in mice^8^. Besides their known functions as vascular sentinel cells, considerable evidence points to a role for platelets in innate and adaptive immunity, engaging with various immune cells^9,10^. Platelets harbour membrane receptors able to detect pathogen- and danger-associated molecular patterns (PAMPs and DAMPs), such as Toll-like receptors (TLRs)^11,12^, rendering them as a functional immune cell.

The elimination of obsolete, damaged, or infected cells via different cell death signalling pathways is critical for the normal development and homeostasis of multicellular organisms. Over the years, multiple cell death signalling pathways have been uncovered. Seminal discoveries using genetically-modified mouse models and pharmacological inhibitors of BCL-2 pro-survival proteins have elucidated that megakaryocytes and platelets contain a functional BAK/BAX mediated intrinsic apoptosis pathway that regulates platelet lifespan^13–17^. The extrinsic apoptosis pathway can be activated by death ligands binding to their cognate death receptors such as Fas or tumour necrosis factor receptor (TNFR)-1, which results in activation of Caspase-8, the essential executioner of the pathway. Studies of genetically-modified mice have demonstrated that megakaryocytes, but not platelets, express Fas receptor on their surface, with FasL triggering extrinsic apoptosis in megakaryocytes^18^. While platelets express Caspase-8, whether or not extrinsic apoptosis can be triggered in platelets remains unknown^8^. Defined as a caspase-independent lytic cell death pathway and involving pro-inflammatory responses, necroptosis is a programmed pathway of necrosis and can be activated via TNFR signalling, TLRs, intracellular DNA and RNA sensors and viral infection^19^. Unlike apoptosis, necroptosis is not immunologically silent^20,21^. Upon activation of these receptors, necroptosis signalling is initiated in scenarios where the extrinsic apoptosis regulator, Caspase-8, and E3 ubiquitin ligases of the Inhibitor of Apoptosis (IAP) family are inhibited or depleted. The apical kinase, Receptor-interacting serine/threonine protein kinase 1 (RIPK1) is activated by autophosphorylation^22^, which enables engagement and activation of RIPK3 kinase within a cytoplasmic high molecular weight assembly termed the necrosome^23^. Necrosomal recruitment and RIPK3-mediated phosphorylation of the terminal effector in the pathway, the pseudokinase MLKL, leads to its activation, oligomerization, and trafficking to, and compromise of the plasma membrane to induce cell death^24–28^. Phosphorylated MLKL forms oligomers that translocate to intracellular membranes and the plasma membrane, inducing membrane rupture, and the release of DAMPs^29^. Necroptosis has been implicated in mediating various diseases, including systemic inflammation, ischemic reperfusion injury and neurodegeneration^20,30,31^.

It has been shown that RIPK3 has a role in regulating haemostasis and thrombosis; tail bleeding and occlusion times in a model of arteriole thrombosis were significantly extended in *Ripk3*^−/−^ mice compared with their wild-type counterparts^32^. Interestingly, platelet necrosis has also been suggested to play a role in the procoagulant platelet response, with platelets exhibiting sustained levels of cytosolic Ca^2+^ and loss of membrane integrity^33^. In contrast to platelets, the role of necroptosis proteins in megakaryocytes has not been investigated, and it is unknown whether necroptotic cell death might contribute to or diminish platelet production. In support of megakaryocytes undergoing necroptosis negatively influencing platelet production, mice homozygous for a D139V substitution of MLKL (*Mlkl*^*D139V*^), which conferred RIPK3-independent activation of necroptosis, developed lethal postnatal inflammation and analysis of postnatal (P) pups at day 3 revealed attenuated platelet numbers^31^. Furthermore, adult mice heterozygous for *Mlkl*^*D139V*^ displayed a significant reduction in platelet numbers in response to stress triggered by 5FU and post irradiation compared to control counterparts^31^. Conversely, a lytic model has been described where an inflammatory stimulus triggered megakaryocyte rupture yielding elevated platelet counts during acute platelet needs^6^. Such a lytic model of platelet release suggests that megakaryocytes might also generate platelets via necroptotic cell death. The dichotomy between the above observations underscored the need to further examine the necroptotic pathway in platelet production.

To investigate the role of the necroptosis signalling cascade in the megakaryocytic lineage, we used genetically-modified mouse models with conditional deletion of *Caspase-8* and *Mlkl* in the megakaryocytic lineage. For the first time, we show that megakaryocytes possess a functional necroptosis signalling pathway. Analyses at steady state and post antibody-mediated thrombocytopenia revealed that platelet production was normal in the absence of MLKL, however, platelet activation and haemostasis were impaired. Additionally, we observed transient protection from the thrombocytopenia that arises from lipopolysaccharide (LPS)-induced platelet consumption and clearance when *Caspase-8* was deleted in the megakaryocytic lineage. Together, our data provide support for a role for extrinsic apoptosis, rather than necroptosis, as the cause of thrombocytopenia in innate immune responses to bacterial pathogens. We conclude that MLKL plays a role in regulating platelet function and haemostasis and that necroptosis signalling in megakaryocytes is dispensable for platelet production.

## Results

### Megakaryocytes harbour a functional necroptosis signalling pathway

**Fig. 1:**
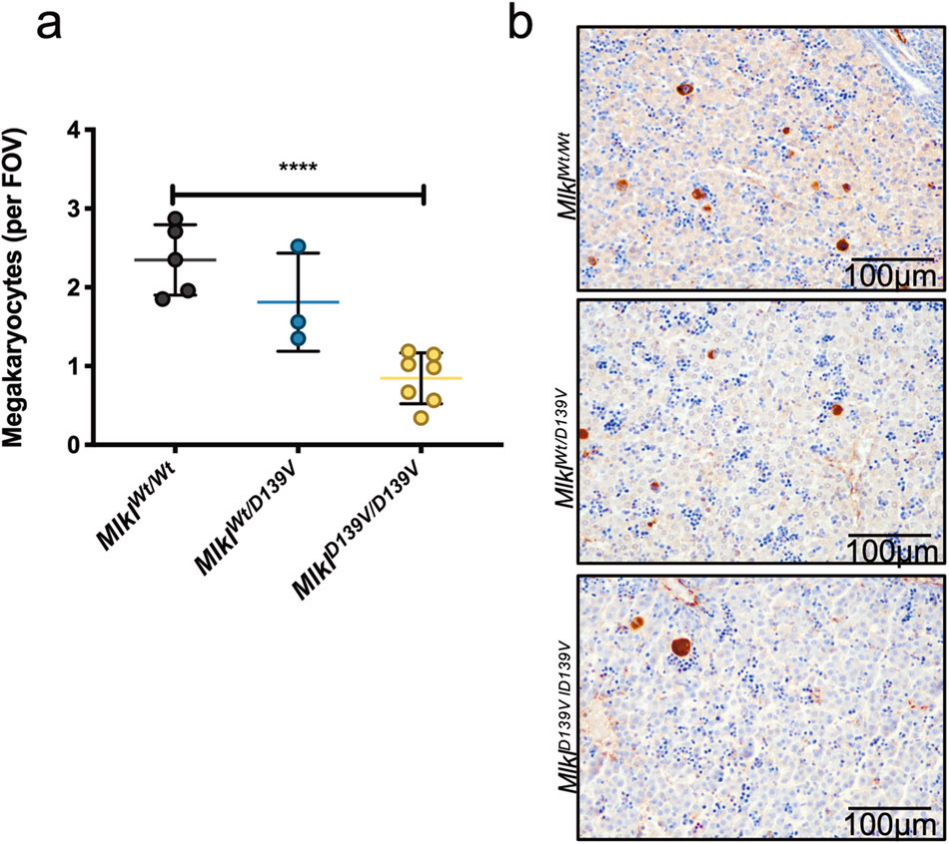
Liver megakaryocyte numbers are deficient in newborn *Mlkl*^*D139V/D139V*^ mice. **a** vWF-positive liver megakaryocyte numbers per field of view (FOV) in P2 *Mlkl*^*Wt/Wt*^ (*n* = 5)*, Mlkl*^*Wt/D139V*^ (*n* = 3) and *Mlkl*^*D139V/D139V*^ (*n* = 7) mice. **b** Representative vWF staining of liver megakaryocytes (brown) from *Mlkl*^*Wt/Wt*^*, Mlkl*^*Wt/D139V*^ and *Mlkl*^*D139V/D139V*^ mice. Data represent mean ± s.d. Student’s unpaired *t*-test, ****P < 0.0001.

Mice homozygous for the *Mlkl*^*D139V*^ allele, which confers constitutive killing activity upon MLKL, the essential effector of necroptotic cell death^31^, develop thrombocytopenia by day P3 with lethal postnatal inflammation of the salivary glands and pericardium^31^. The majority of megakaryocytes reside in the liver at P1–P3^34^ and we assessed the number of liver megakaryocytes in P2 *Mlkl*^*D139V/D139V*^, *Mlkl*^*Wt/D139V*^ and *Mlkl*^*Wt/Wt*^ control mice to investigate whether the thrombocytopenia could be due to reduced numbers of megakaryocytes. Strikingly, the number of von Willebrand factor (vWF)-positive megakaryocytes in P2 *Mlkl*^*D139V/D139V*^ livers were significantly decreased compared to *Mlkl*^*Wt/Wt*^ control livers (*p* < 0.0001) (Fig. 1a, b, Supplementary Fig. 1). Based on these observations, we hypothesised that direct activation of necroptosis in megakaryocytes is the underlying cause of megakaryocytic cell death.

**Fig. 2:**
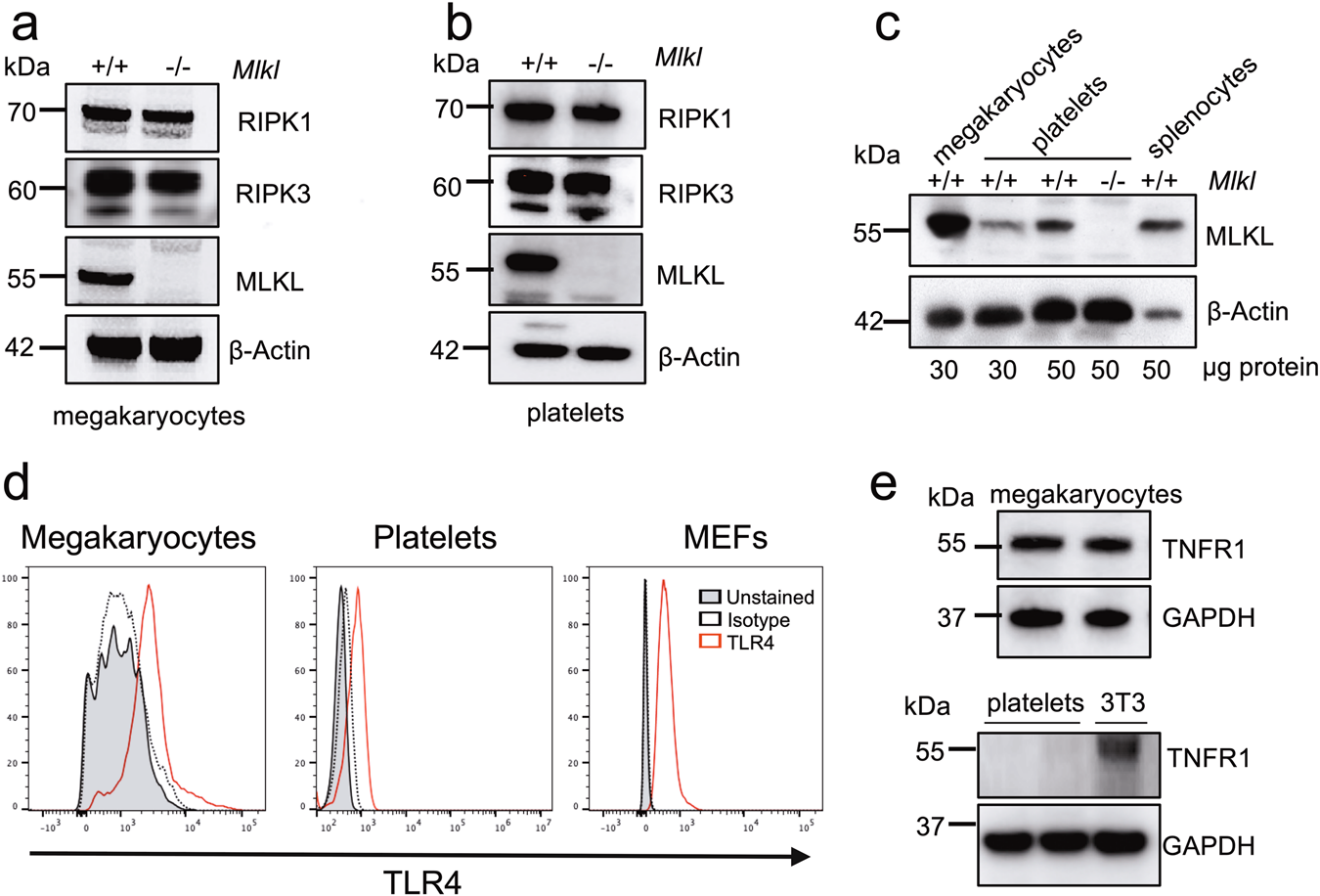
Expression of necroptosis signalling pathway proteins in the megakaryocytic lineage. **a**, **b** Western blot analysis of protein lysates from WT or *Mlkl*^*−/−*^ BM-derived cultured megakaryocytes or purified blood platelets probed for RIPK1, RIPK3 and MLKL. β-Actin was used as a loading control**. c** Western blot analysis of protein lysates from megakaryocytes, platelets and splenocytes with the indicated genotypes. WT platelets have increasing protein concentration loaded and probed for MLKL. β-Actin was used as a loading control. **d** TLR4 surface expression was determined by flow cytometry on BM-derived CD41^+^ megakaryocytes and purified blood platelets from WT mice. Mouse embryonic fibroblasts (MEFs) were included as a positive control. Representative histograms are shown. **e** Western blot analysis of protein lysates from WT BM-derived cultured megakaryocytes, WT purified blood platelets or 3T3 cells for TNFR1. GAPDH was used as a loading control.

**Fig. 3:**
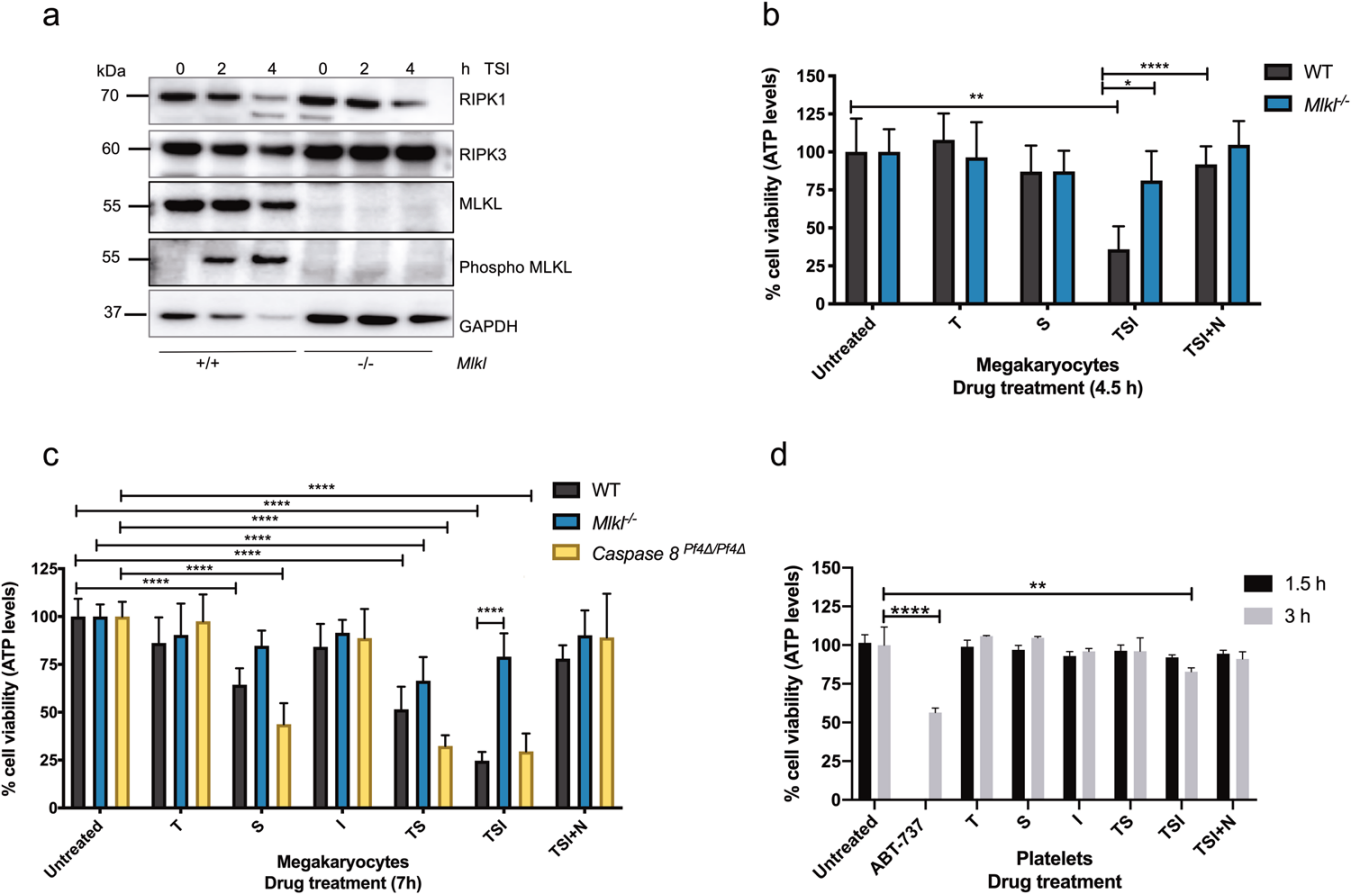
Megakaryocytes, but not platelets, undergo TSI-induced necroptosis. **a** Western blot analysis of protein lysates from WT or *Mlkl*^−/−^ BM-derived cultured megakaryocytes untreated or treated with TSI (TNF, 100 ng/ml; Smac mimetic, 0.5 µM; and IDN-6556, 5 µM) for 2 h and 4 h at 37 °C and probed for the indicated proteins. GAPDH was used as a loading control. **b**, **c** Cell viability in BM-derived cultured megakaryocytes from WT, *Mlkl*^−/−^ and *Caspase-8*^*Pf4Δ/Pf4Δ*^ mice untreated or treated with the indicated agents (TNF, 100 ng/ml (T); Smac mimetic, 0.5 µM (S); IDN-6556, 5 µM (I); Necrostatin-1, 50 µM (N). ATP levels in megakaryocytes were measured 4.5 h in (**b**) or 7 h in (**c**) after treatment at 37 °C using the Cell Titer-Glo assay relative to untreated control, *n* = 3 independent experiments per genotype. **d** Cell viability in purified blood platelets from WT mice untreated or treated with the indicated agents (same concentration as in (**b**, **c**) and 1 µM ABT-737). ATP levels in platelets were measured 1.5 and 3 h after treatment at 37 °C using the Cell Titer-Glo assay relative to untreated control (1.5 h treated compared to 1.5 h untreated, 3 h treated compared to 3 h untreated). *n* = 3 biological replicates, data represent mean ± s.d. One-way ANOVA with the Dunnett multiple comparison test relative to control untreated WT, *Mlkl*^−/−^ or *Caspase-8*^*Pf4Δ/Pf4Δ*^, or where otherwise indicated. *P < 0.05, **P < 0.005, ***P < 0.001, ****P < 0.0001.

We first examined the expression of RIPK1, RIPK3, MLKL, TNFR1 and TLR4 proteins in washed platelets and mature megakaryocytes purified from cultures of BM cells^25^. Megakaryocytes and platelets from *Mlkl*^−*/*−^ mice were included as negative controls for MLKL expression and wild-type splenocytes as a positive control. By western blotting, we could readily detect all components of the necroptosis signalling pathway; RIPK1, RIPK3 and MLKL in lysates of wild-type platelets and megakaryocytes (Fig. 2a–c). It was evident that MLKL was highly expressed in megakaryocytes, with a more prominent signal observed than that in platelets and splenocyte controls (Fig. 2c). In vitro, various ligands for TNFRs or TLRs can trigger necroptosis. Flow cytometric analysis using TLR4 antibodies revealed the presence of TLR4 on the surface of wild-type platelets and BM-derived megakaryocytes (Fig. 2d). Consistent with previous reports, the classical receptor for stimulation of necroptosis signalling, TNFR1, was detected by Western blotting in megakaryocytes^35^, but not at marked levels in platelets (Fig. 2e)^36^. These observations suggested that while megakaryocytes and platelets express the cytoplasmic components of the necroptosis pathway, only megakaryocytes are capable of responding to the classical necroptosis pathway agonist TNF. For TNFR1-stimulated necroptosis, this can occur with TNF, concomitant depletion of cIAPs using a Smac-mimetic (small molecule IAP antagonist compound), such as Compound A, and pharmacological inhibition or genetic deletion of Caspase-8^23,37,38^. We, therefore, tested the effect of TNF combined with Smac mimetic and pan-Caspase inhibitor (IDN-6556/Emricasan) (hereafter referred to as TSI) on both cell types^39^. Phospho-MLKL was readily detected in western blots of wild-type mature megakaryocytes derived from BM cultures at 2 h and 4 h post TSI treatment (Fig. 3a), demonstrating activation of the executioner protein in the necroptosis signalling cascade. Moreover, TSI treatment triggered phosphorylation of upstream RIPK1 and RIPK3 (Supplementary Fig. 2a). Megakaryocyte viability was then assessed by the Cell Titer Glo assay, measuring ATP abundance 4.5- and 7-h post TSI treatment (Fig. 3b, c). Treatment with TSI resulted in substantial necroptosis induced cell death in wild-type megakaryocytes but not *Mlkl*^*−/−*^ megakaryocytes, consistent with this death being necroptotic in nature (Fig. 3b, c). This effect was directly attributable to caspase inhibition, because a different pan-Caspase inhibitor QVD-OPh yielded a similar result (Supplementary Fig. 2b). Loss of viability was observed in wild-type megakaryocytes at 4.5 h post TSI and became more pronounced at 7 h post treatment, while *Mlkl*^−*/*−^ megakaryocytes were resistant to necroptosis at both timepoints (Fig. 3b, c). Moreover, treatment of wild-type megakaryocytes with TSI and concomitant addition of Necrostatin-1, a RIPK1 inhibitor, rescued wild-type megakaryocytes from TSI induced necroptosis (Fig. 3b, c). Treatment with TNF and Smac mimetic (TS) for 7 h (in the absence of caspase inhibition) triggered apoptotic cell death, where wild-type and *Mlkl*^*−/−*^ megakaryocytes exhibited reduced viability to a similar degree (Fig. 3c). Furthermore, we included mature BM derived megakaryocytes from *Caspase-8*^*Pf4Δ/Pf4Δ*^ mice^18^, to test the hypothesis that they would be sensitised to necroptosis using TS in the absence of a chemical caspase inhibitor. As expected, *Caspase-8*^*Pf4Δ/Pf4Δ*^ megakaryocytes treated with TS were sensitized to necroptosis, notably with viability levels comparable to wild-type and *Caspase-8*^*Pf4Δ/Pf4Δ*^ megakaryocytes treated with TSI for 7 h (Fig. 3c).

We next assessed if TSI could initiate necroptosis in murine platelets. As anticipated since TNFR1 was not detected at marked levels in platelets, necroptosis was not triggered in wild-type platelets treated with TSI for 1.5 h at 37 °C (Fig. 3d). Murine platelets quickly lose viability during in vitro storage at 37 °C^40^. After 3 h at 37 °C untreated platelets displayed ~30% loss in cell viability compared to untreated platelets at 1.5 h (Supplementary Fig. 2c). As previously reported, the BH3-mimetic, ABT-737 notably reduced platelet viability^40^ (Fig. 3d), but TSI treatment had only a marginal, albeit significant, effect on viability when compared to untreated platelets at 3 h (Fig. 3d, Supplementary Fig. 2c). Collectively, the results indicated that megakaryocytes, but not platelets undergo necroptosis triggered via TNFR1 signalling.

### Morphological differences of megakaryocytes undergoing necroptosis and apoptosis

**Fig. 4:**
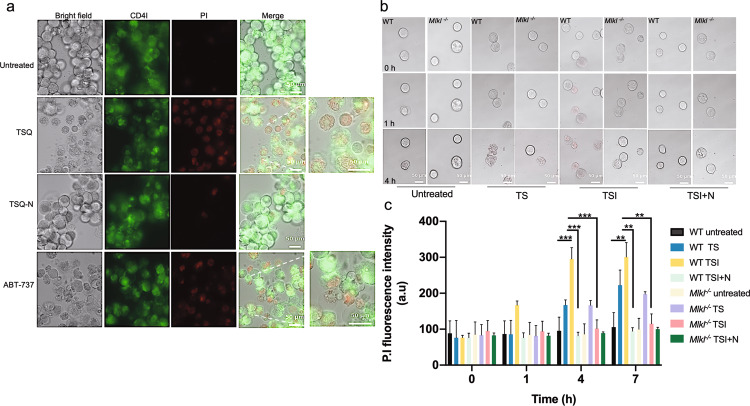
Morphology of megakaryocytes in response to TNF-induced necroptosis and apoptosis. **a** Representative time-lapse confocal images from WT BM-derived cultured megakaryocytes treated with TNF (100 ng/ml, T), Smac mimetic (0.5 µM, S), QVD-OPh (50 µM, Q), Necrostatin-1 (50 µM, N), and ABT-737 (5 µM). Megakaryocytes were labelled with CD41 conjugated to Alexa-488 (green) and PI (propidium iodide, red), Bar 50 µm. Cells were maintained on the stage within a humidified chamber at 37 °C and 5% CO_2_ for 7 h. Images were acquired on a Ziess live cell Axio inverted modular microscope for live cell imaging every 10 min driven by AxioVision v4.8 software. **b** Representative time-lapse confocal images from WT and *Mlkl*^−/−^ BM-derived cultured megakaryocytes treated with the indicated agents: TNF (100 ng/ml, T), Smac mimetic (0.5 µM, S), IDN-6556 (5 µM, I), Necrostatin-1 (50 µM, N) at the indicated time points (hours) at 37 °C, images acquired as in (**a**). **c** Quantification of PI from representative time-lapse video microscopy at the indicated time points (hours). PI fluorescence intensity was quantified using a custom macro in Fiji software. *n* = 3 independent experiments, data presented as mean ± SEM, One-way ANOVA with the Bonferroni multiple comparison test, **P < 0.005; ***P < 0.001.

To compare the morphology of megakaryocytes undergoing necroptosis relative to apoptosis and to ascertain whether necroptosis could influence proplatelet formation or platelet release from megakaryocytes, we carried out live cell time lapse confocal microscopy. Gradient-purified mature BM-derived wild-type megakaryocytes were treated with TSQ (TNF; Compound A, Smac mimetic; and QVD-OPh, pan caspase inhibitor) to trigger necroptosis. This was carried out in parallel with wild-type megakaryocytes treated with ABT-737, previously shown to trigger BAK/BAX mediated intrinsic apoptosis, resulting in robust cell death^14,16^, (Fig. 4a). Representative still images at 7 h post TSQ treatment demonstrated that wild-type megakaryocytes displayed characteristic features of necroptosis (Fig. 4a), such as swelling of cells and loss of membrane integrity, which was blocked by Necrostatin-1 treatment. Conversely, wild-type megakaryocytes treated with ABT-737 displayed distinctive features of apoptosis, such as membrane blebbing, cell shrinkage and the formation of apoptotic bodies (Fig. 4a), similar to what has previously been observed in vitro post genetic deletion of *Bcl-x* in megakaryocytes^14^. Representative still images of megakaryocytes labelled with CD41 in green and the dead cell marker, propidium iodide (PI), in red at 7 h post TSQ demonstrated that wild-type megakaryocytes also became PI positive in addition to the characteristic features of necroptosis (Fig. 4a). Necroptosis was blocked by Necrostatin-1 treatment, where megakaryocytes largely remained PI negative and maintained their morphology (Fig. 4a). Conversely, wild-type megakaryocytes treated with ABT-737 became PI positive and displayed distinctive features of apoptosis, such as membrane blebbing, cell shrinkage and the formation of apoptotic bodies (Fig. 4a)^14,18^.

Next, we performed live cell time lapse confocal microscopy to compare necroptosis and apoptosis in gradient-purified wild-type and *Mlkl*^*−*/−^ megakaryocytes. Cells were treated with TSI to trigger necroptosis or TS to trigger extrinsic apoptosis and then stained with PI to monitor the extent of cell death. Representative images at 1 h and 4 h post treatment revealed that necroptosis was a quick process in megakaryocytes, evident as early as 1 h post TSI treatment, while apoptosis triggered by TS was slower and evident by 4 h (Fig. 4b). Quantification of PI fluorescence intensity from time-lapse images demonstrated a significant increase in PI fluorescence intensity at 4 h and 7 h post TSI in wild-type megakaryocytes relative to untreated wild-type megakaryocytes, and a significant reduction in PI fluorescence intensity in the presence of necrostatin-1 (Fig. 4b, c). Consistent with this being necroptotic death, *Mlkl*^*−*/−^ megakaryocytes were refractory to necroptosis triggered by TSI, however, as expected were as sensitive to TS induced apoptosis as wild-type megakaryocytes (Fig. 4b, c and Supplementary Fig. 3). We did not observe pro-platelet formation or platelet shedding in megakaryocytes post-TSI treatment (when imaged every 10 min for a 7-h period), indicating that necroptosis triggered by TNF signalling in megakaryocytes does not promote platelet formation.

### Steady state and stress-induced platelet production are normal in the absence of *Mlkl*

**Fig. 5:**
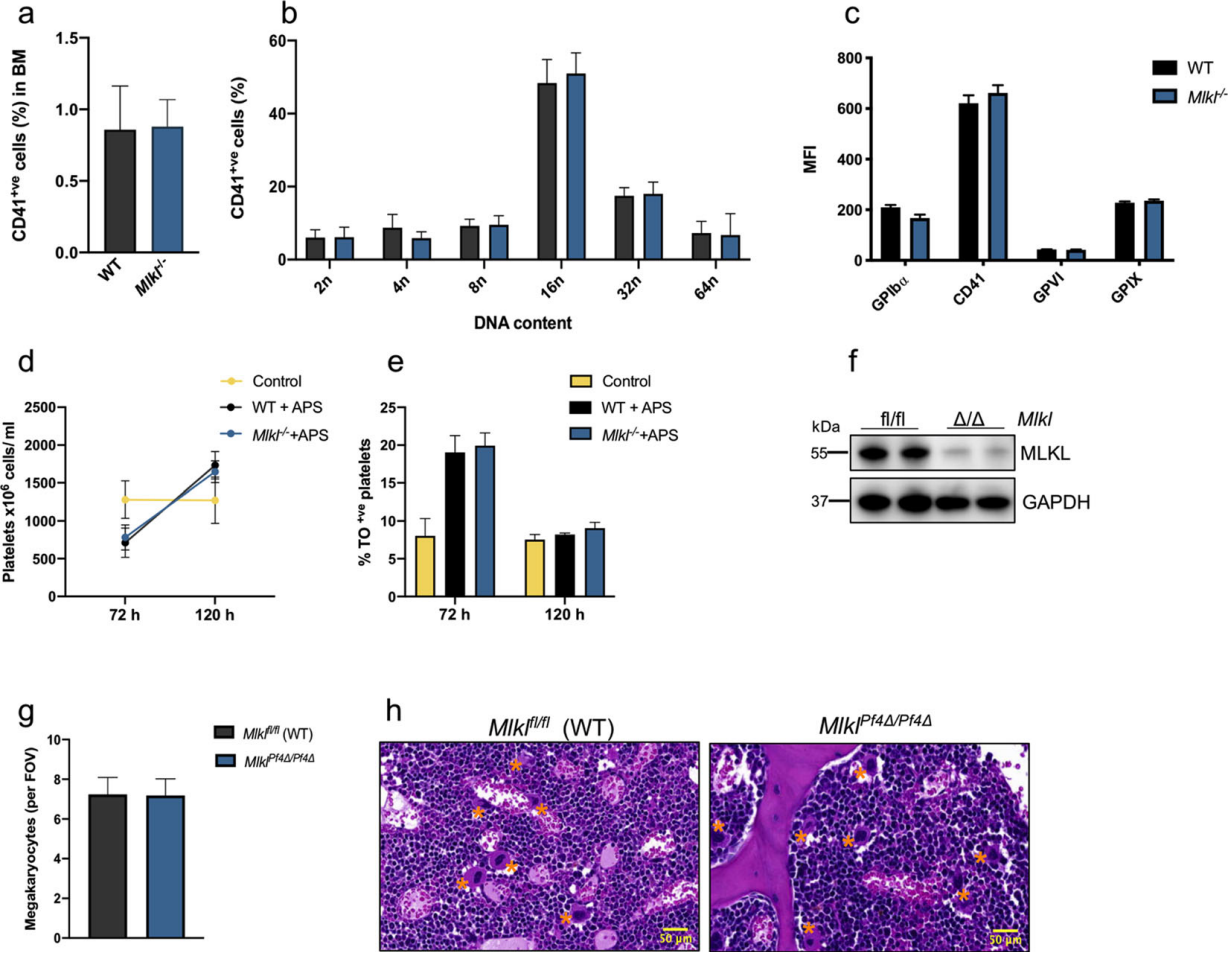
Steady state and stress-induced platelet production is normal in the absence of *Mlkl*. **a** Percentage of CD41^+^ cells (megakaryocytes) in WT and *Mlkl*^−/−^ BM assessed via flow cytometry. WT *n* = 6 and *Mlkl*^−/−^ *n* = 6. **b** Ploidy distribution profile of CD41/PI-positive BM cells in WT and *Mlkl*^−/−^ mice as determined by flow cytometry, WT *n* = 6 and *Mlkl*^−/−^ *n* = 6. **c** Platelet surface receptor expression by mean fluorescence intensity (MFI) of GPIX, GPIbα, GPVI and CD41 were assessed on purified platelets from WT and *Mlkl*^−/−^ mice by flow cytometry, WT *n* = 3 and *Mlkl*^−/−^ *n* = 3. **d** Platelet counts and **e** percentage of thiol orange (TO)-positive reticulated platelets in WT and *Mlkl*^*−/−*^ mice post anti-platelet serum (APS) administration. Cohorts of mice were bled at the indicated time points, 72 h control *n* = 4, WT + APS *n* = 5, *Mlkl* ^−/−^ + APS *n* = 4; 120 h control *n* = 4, WT + APS *n* = 4 and *Mlkl* ^−/−^ + APS *n* = 5. **f** Representative western blot analysis of protein lysates from *Mlkl*^*fl/fl*^ or *Mlkl*^*Pf4Δ/Pf4Δ*^ purified blood platelets probed for MLKL. GAPDH was used as a loading control. **g** Megakaryocyte numbers per FOV in H&E-stained sections of BM from *Mlkl*^*Pf4Δ/Pf4Δ*^ and *Mlkl*^*fl/fl*^ control mice, *n* = 5 per genotype. **h** Representative images of H&E stained sternal sections from *Mlkl*^*Pf4Δ/Pf4Δ*^ and *Mlkl*^*fl/fl*^ control mice. Megakaryocytes are indicated by asterisks. *n* = 5 mice per genotype. Data are presented as mean ± s.d, student’s unpaired *t*-test, no significant differences.

It has previously been shown that platelet counts and megakaryocyte numbers in the BM and spleen are unperturbed in *Mlkl*^*−*/−^ mice at steady state, suggesting that megakaryopoiesis is normal in these mice in the absence of stress^25,41,42^. To further characterise the outcomes of *Mlkl* loss in the megakaryocytic lineage, we assessed the percentage of megakaryocytes in BM from wild-type control and *Mlkl*^*−/−*^ mice and their ploidy profile by flow cytometry using the megakaryocyte and platelet specific marker CD41 (Fig. 5a, b). The percentage of CD41^+^ cells in BM from *Mlkl*^−*/−*^ mice was similar to that in wild-type control mice (Fig. 5a) and the ploidy profile was normal (Fig. 5b). Furthermore, surface receptor expression of major platelet receptors CD41, GPIX, GPIbα and GPVI did not differ between wild-type control animals and *Mlkl*^*−/−*^ mice (Fig. 5c), confirming that the expression of platelet receptors was unperturbed in *Mlkl*^−*/−*^ mice at steady state.

The gradual loss of quality in stored platelet concentrates, marked by biochemical, morphological and functional changes in vitro, is known as the platelet storage lesion (PSL)^43^, and is a major impediment to storing platelets for clinical use. We have previously shown that inhibition of intrinsic apoptosis by deletion of *Bak/Bax* in platelets, does not improve the PSL^40^. To examine the role of necroptosis in the development of the PSL we next mimicked the standard storage conditions used for human platelet concentrates, storing murine platelets in modified Tyrode-HEPES buffer containing plasma at 22 °C with agitation (60 rpm), for a total of 5 days. As expected, wild-type control platelets gradually lost viability, when assessed for ATP abundance (Supplementary Fig. 4a) and shed surface receptors GPVI (Supplementary Fig. 4b) and GPIbα (Supplementary Fig. 4c). Similar to wild-type platelets, the platelets lacking *Mlkl* exhibited the same loss of viability and receptor shedding, indicating that inhibition of necroptosis is unlikely to improve the PSL.

Next, we examined the ability of wild-type and *Mlkl*^*−/−*^ mice to recover from acute thrombocytopenia induced by a single dose of anti-platelet serum (APS). Previous studies have shown that 24 h after administration of APS, platelet counts are reduced to almost undetectable levels^40^. Subsequently, wild-type and *Mlkl*^*−/−*^ animals mounted a robust recovery of similar magnitude and kinetics, assessed by the percentage of reticulated thiazole orange (TO) positive platelets, and platelet counts, with platelet counts exceeding baseline levels by 5 days post-APS injection (Fig. 5d, e). Together, the data demonstrated that MLKL does not play an obligate role in emergency megakaryopoiesis.

We next generated mice in which deletion of *Mlkl* was regulated under control of the platelet factor 4 promoter (*Pf4*). At weaning, *Mlkl*^*Pf4Δ/Pf4Δ*^ mice were present at the expected Mendelian ratios and developed normally. Adult *Mlkl*^*Pf4Δ/Pf4Δ*^ mice were healthy and fertile. Western blot analysis confirmed that MLKL was efficiently deleted in platelets (Fig. 5f) and megakaryocytes (Fig. 7a). Platelet count and other peripheral blood counts in *Mlkl*^*Pf4Δ/Pf4Δ*^ mice were comparable to wild-type littermate controls (Table 1), akin to normal platelet counts in *Mlkl*^−/−^ mice^25,41^. Megakaryocyte numbers were enumerated from haematoxylin and eosin (H&E) stained sternal sections and revealed no change in number or morphology compared to floxed control mice (Fig. 5g, h). Overall, these data demonstrate that conditional deletion of *Mlkl* in the megakaryocytic lineage does not influence steady state platelet production.

**Table 1:** Peripheral blood counts from 7- to 10-week-old (*Caspase-8*^*fl/fl*^ *Mlkl*^*fl/fl*^ (wild-type), *Caspase-8*^*Pf4Δ/Pf4Δ*^*, Mlkl*^*Pf4Δ/Pf4Δ*^*, Caspase-8*^*Pf4Δ/Pf4Δ*^ *Mlkl*^*Pf4Δ/Pf4Δ*^) female and male mice.

	*Caspase-8*^*fl/fl*^ *Mlkl*^*fl/fl*^ (*n* = 16)	*Caspase-8*^*Pf4Δ/Pf4Δ*^ (*n* = 7)	*Mlkl*^*Pf4Δ/Pf4Δ*^ (*n* = 8)	*Caspase-8*^*Pf4Δ/Pf4Δ*^ *Mlkl*^*Pf4Δ/Pf4Δ*^ (*n* = 8)
Platelets, × 10^6^/mL	1066 ± 176.9	968 ± 186	1087 ± 103.6	1138 ± 118.2
MPV, femtoliters	5.4 ± 0.4	4.9 ± 0.1^∗∗^	5.8 ± 0.3^*^	5.6 ± 0.2
PDW, % measurement	50.7 ± 5.7	50.6 ± 7.2	49.7 ± 2.1	49.9 ± 4.2
Hematocrit, %	50.7 ± 2.1	49.8 ± 2.3	50.0 ± 0.6	51.6 ± 2.0
Erythrocytes, × 10^9^/mL	10.4 ± 0.3	10.5 ± 0.5	10.1 ± 0.1^∗^	10.5 ± 0.3
Leukocytes, × 10^6^/mL	8.4 ± 1.5	9.9 ± 2.2	9.7 ± 0.9	7.9 ± 1.0
Neutrophils, × 10^6^/mL	0.9 ± 0.6	1.1 ± 0.4	1.0 ± 0.4	0.5 ± 0.1
Lymphocytes, × 10^6^/mL	7.1 ± 1.1	8.4 ± 2.3	8.3 ± 0.9	7.0 ± 0.9
Monocytes, × 10^6^/mL	0.2 ± 0.1	0.2 ± 0.1	0.2 ± 0.1	0.2 ± 0.1
Eosinophils, × 10^6^/mL	0.1 ± 0.1	0.1 ± 0.1	0.1 ± 0.0	0.1 ± 0.0

Data represent mean ± SD, one-way ANOVA with Dunnett’s multiple comparison test. Data are compared with *Caspase-8*^*fl/fl*^ *Mlkl*^*fl/fl*^ (wild-type) mice.

*MPV* mean platelet volume, *PDW* platelet distribution width.

*P < 0.05. **P < 0.005.

### Unstable thrombus formation in *Mlkl*^*−/−*^ mice

**Fig. 6:**
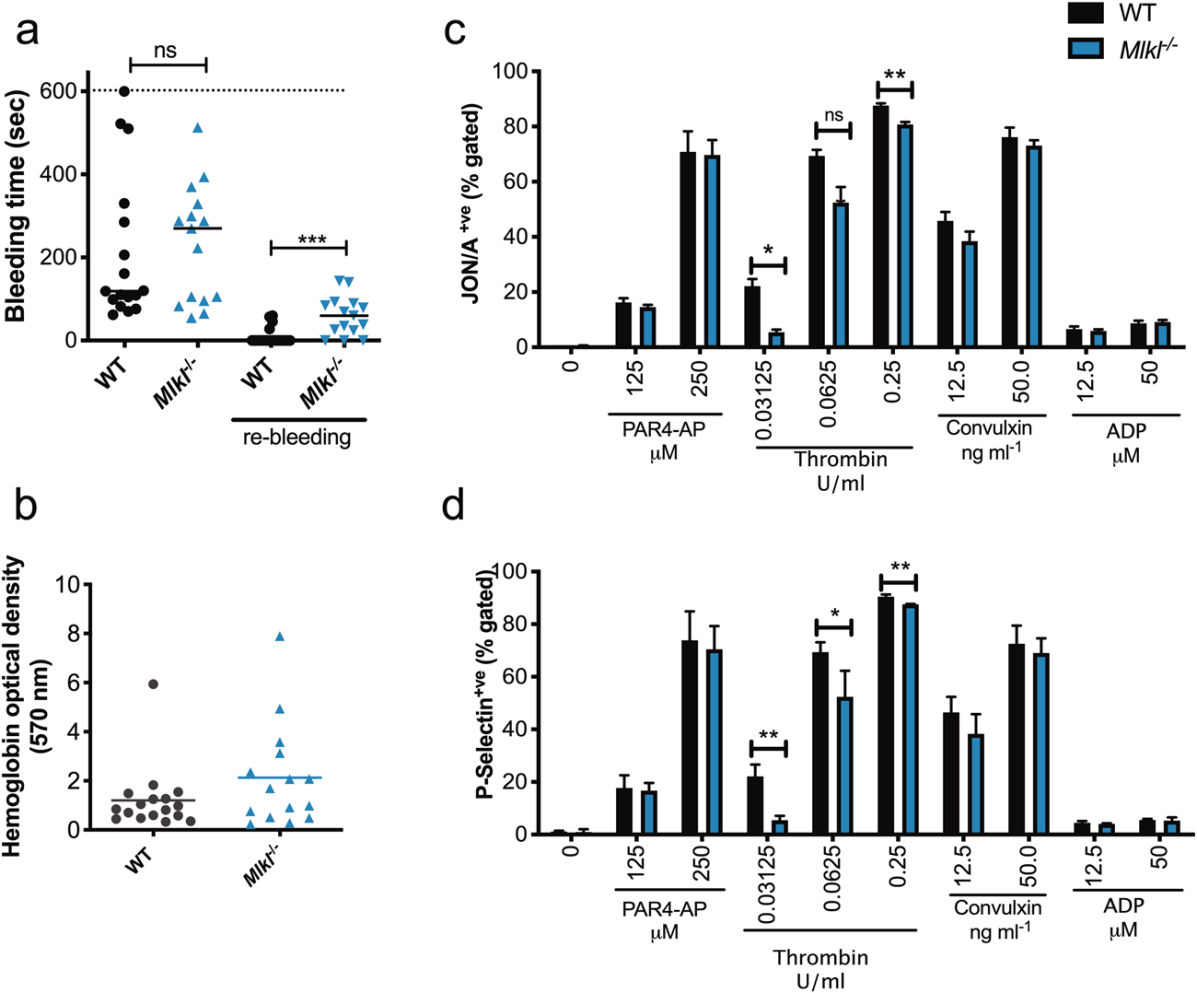
Loss of *Mlkl* influences platelet activation and results in impaired haemostasis. **a** Tail bleeding time into 37 °C saline (3-mm tail amputation) in WT (*n* = 17) and *Mlkl*^−/−^ (*n* = 15) mice. Maximal time was set as 600 s. The bleeding time was determined as the time from the tail amputation to the moment the blood flow stopped for more than 2 min. Following cessation of bleeding for 2 min, the incision was monitored for a further 2 min, and bleeding within this period classified as re-bleeding. The length of re-bleeding time is not included in the two first groups, but shown separately as “re-bleeding”. Each symbol represents an individual mouse. Data is combined from two independent experiments. Male and female mice were included. Data are presented as median. **b** Blood loss assessed by haemoglobin levels in WT and *Mlkl*^−/−^ mice; WT *n* = 17 and *Mlkl*^−/−^ *n* = 15. Agonist-induced platelet activation in WT and *Mlkl*^−/−^ platelets determined by **c** integrin activation (JON/A) or **d** P-selectin positive platelets by flow cytometry, statistically-significant differences are shown relative to WT control platelets at the indicated concentration of agonist. Platelets were washed and counts adjusted before 20 min incubations with ADP (37 °C), Convulxin (room temperature (RT)), PAR4-AP (RT) and Thrombin (RT) at the indicated concentrations, *n* = 3–5 mice per genotype. Data are presented as mean ± SEM, student’s unpaired *t*-test, *P < 0.05; **P < 0.005; ***P < 0.001, ns; not significant.

Previously, it has been shown that MLKL’s upstream activator, the RIPK3 kinase, plays a role in regulating thrombosis and haemostasis^32^: *Ripk3*^−/−^ mice displayed prolonged bleeding times and reduced degranulation in response to thrombin and thromboxane A2 analogue. RIPK3 has been attributed other signalling functions in addition to its function as a necroptosis effector, and accordingly we sought to establish whether the previous reports related to RIPK3’s necroptotic functions in haemostasis by examining *Mlkl-*deficient mice. To investigate the role of MLKL in haemostasis, we assessed bleeding times into 37 °C saline, after 3-mm tail amputations in *Mlkl*^−/−^ and wild-type mice. *Mlkl*^−/−^ mice exhibited modest, but statistically significant, prolonged tail re-bleeding time(s) compared to wild-type mice, assessed over a 10-min period, indicating unstable thrombus formation (Fig. 6a). However, blood loss (haemoglobin levels) did not differ between the two groups (Fig. 6b). This result demonstrated that MLKL plays a role in thrombus formation in vivo.

We next analysed platelet signalling pathways downstream of classical platelet activation by flow cytometry, assessing integrin αIIbβ3 (CD41/CD61) activation (JON/A antibody) and degranulation of α-granules by P-selectin exposure. There was a significant reduction in integrin αIIbβ3 activation in *Mlkl*^−/−^ and *Mlkl*^*Pf4Δ/Pf4Δ*^ platelets in response to thrombin (Fig. 6c, Supplementary Fig. 4d), and degranulation assessed by P-selectin exposure (Fig. 6d, Supplementary Fig. 4e). Conversely, no difference between genotypes was evident in response to triggering GPVI (Convulxin) or PAR4 (PAR4-AP) receptors (Fig. 6c, d, Supplementary Fig. 4d, e). The response to the agonist, adenosine diphosphate (ADP), which does not promote granule release in this assay, was similar in wild-type and *Mlkl*^−/−^ platelets. We next assessed platelet function in response to thrombin and convulxin by aggregometry. Normal platelet aggregation was evident in *Mlkl*^−/−^ platelets in response to convulxin, however at low doses of thrombin (0.1 U/ml), but not higher concentrations, *Mlkl*^−/−^ platelets aggregated less than wild-type platelets (Supplementary Fig. 4f, g).

Procoagulant platelets are a subpopulation of highly activated platelets that express coagulation-promoting activity by phosphatidylserine (PS) exposure, and membrane ballooning, which may shed as microvesicles. Procoagulant platelets play an important role for clot stabilization during normal haemostasis and high levels of these platelets correlate with transient ischemic attack and stroke^44,45^. Previously, it has been shown that dual agonist-induced platelet procoagulant function was unchanged in *Bak*^−/−^*Bax*^−/−^ platelets suggesting that intrinsic apoptotic machinery in platelets does not play a role in regulating dual agonist-induced platelet procoagulant function^46^. Interestingly, platelet necrosis has been suggested to occur in procoagulant platelets^33^. Therefore, we were prompted to examine the procoagulant response in the absence of functional necroptosis using *Mlkl-*deficient platelets. Wild-type control and *Mlkl*^−/−^ platelets were treated with the dual platelet agonists thrombin and convulxin at three different concentrations (Supplementary Fig. 4h, i). PS externalization was assessed by Annexin V binding and Platelet Δψ_m_ was determined by flow cytometry using the cationic dye TMRM, with *Mlkl*^−/−^ platelets exhibiting normal procoagulant responses (Supplementary Fig. 4h, i). These data indicate that MLKL does not play a role in dual agonist-induced platelet procoagulant responses in vitro.

### Conditional deletion of *Caspase-8* in the megakaryocytic lineage provides transient protection from LPS-induced thrombocytopenia

LPS, also known as endotoxin, is the main structural cell wall component of Gram-negative bacteria. Engagement of LPS by TLR4 can trigger systemic inflammatory responses and tissue injury associated with necrotic cell death^47,48^. The downstream cascade of signalling events includes recruitment of adaptor proteins MyD88, the Toll/IL-1R-containing adaptor molecule (TRIF), and TRIF-related adaptor molecule to elicit both inflammatory cytokine and type I IFN responses^47,49^. Chemical Caspase-8 inhibition or genetic deletion of *Caspase-8* can trigger necroptosis upon TLR4 ligation, which is ablated in RIPK3 or MLKL-deficient cells^41,48,50,51^. Sepsis represents a clinical condition characterised by severe inflammatory responses to infection. Patients with sepsis often have very low platelet counts and administration of LPS in animal models in vivo, can trigger systemic inflammatory responses, promoting thrombocytopenia. LPS-induced production of cytokines such as TNF and IL-6, leads to inflammatory tissue injuries and multiorgan failure^52,53^. The TLR4/MyD88 pathway components are expressed in the megakaryocytic lineage. Mounting evidence demonstrates that LPS stimulates platelet activation, which plays a role in LPS-induced thrombocytopenia and tissue damage, stimulating the accumulation of platelets in the liver and lungs^54–57^.

We next tested if BM-derived megakaryocytes could undergo necroptosis in response to LPS ± caspase inhibitor in vitro. Megakaryocyte viability was not negatively affected 22 h post treatment with 100 ng/ml and 1 µg/ml LPS ± caspase inhibitors QVD-OPh, IDN-6556 or zVAD-fmk when compared to the vehicle control (Supplementary Fig. 5a). CD14 is considered an important partner of TLR4 for ligation of LPS^58,59^. Platelets lack CD14^60^ and to ensure CD14 was present in our megakaryocyte viability assay at sufficient amounts, we added 5% and 10% serum, where soluble CD14 is known to be present^61^, to the assay. Even in the presence of serum, no significant effect on megakaryocyte viability was observed 20 h post addition of 100 ng/ml and 1 µg/ml LPS (Supplementary Fig. 5b). However, reduced viability was detected when 100 ng/ml LPS was combined with IDN-6556 in the presence of 10% serum (Supplementary Fig. 5b). Nevertheless, since no rescue was seen by the addition of the necroptosis inhibitor, necrostatin-1 (Supplementary Fig. 5b), the cell death induced in megakaryocytes by LPS + IDN-6556 in vitro is unlikely to be attributable to necroptosis.

**Fig. 7:**
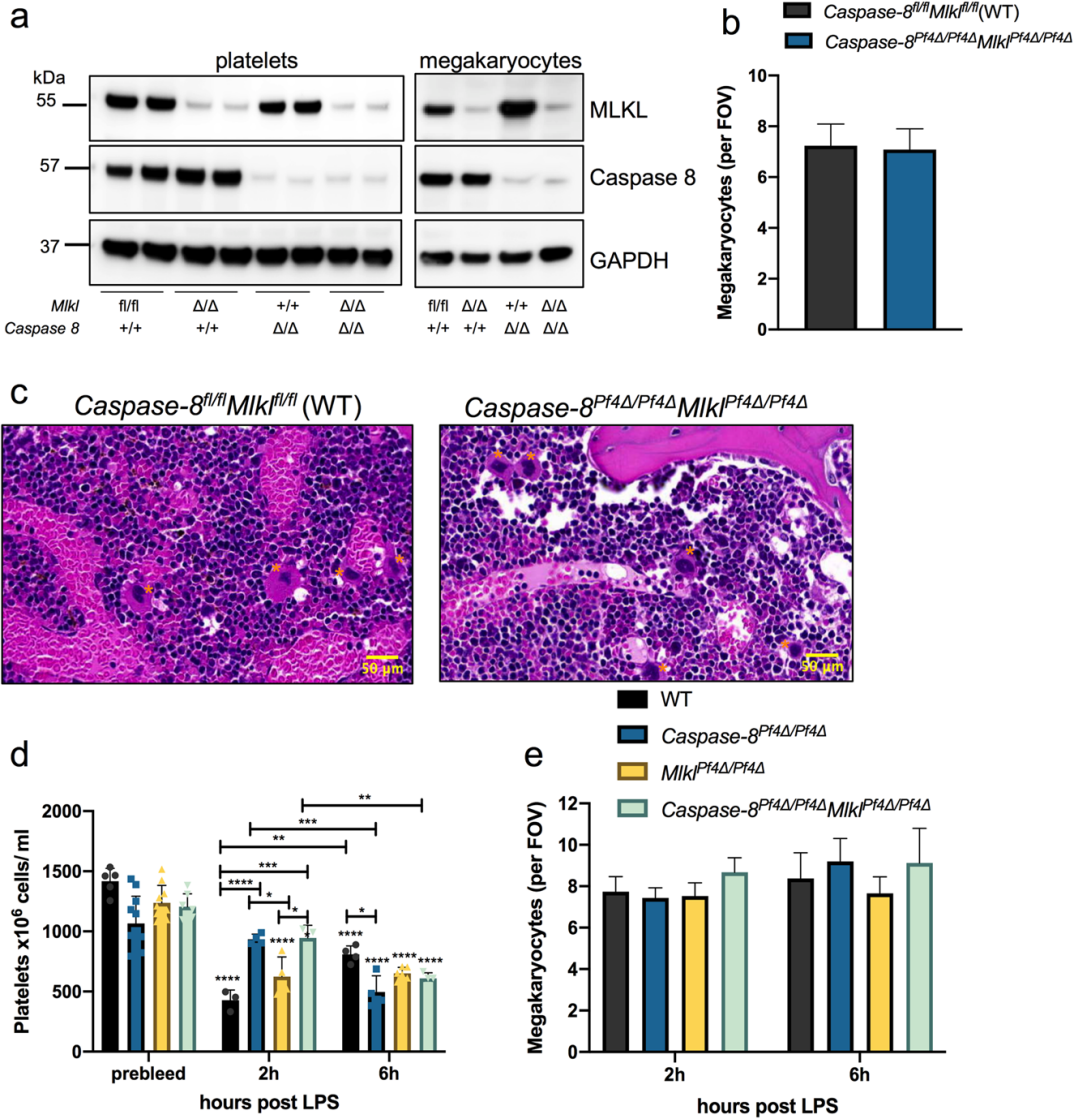
Transient protection from LPS-induced thrombocytopenia in *Caspase-8*^*Pf4Δ/Pf4Δ*^ mice. **a** Western blot analysis of protein lysates generated from floxed control, *Caspase-8*^*Pf4Δ/Pf4Δ*^*, Mlkl*^*Pf4Δ/Pf4Δ*^ and *Caspase-8*^*Pf4Δ/Pf4Δ*^ *Mlkl*^*Pf4Δ/Pf4Δ*^ mice. Purified blood platelets and BSA gradient purified cultured BM megakaryocytes probed with antibodies to Caspase-8 and MLKL. GAPDH was used as a loading control. **b** Megakaryocyte numbers per FOV in H&E-stained sections of BM from *Caspase-8*^*Pf4Δ/Pf4Δ*^*Mlkl*^*Pf4Δ/Pf4Δ*^ (*n* = 5) and *Caspase-8*^*fl/fl*^*Mlkl*^*fl/fl*^ control mice (*n* = 3). **c** Representative images of H&E stained sternal sections from *Caspase-8*^*Pf4Δ/Pf4Δ*^*Mlkl*^*Pf4Δ/Pf4Δ*^ and *Caspase-8*^*fl/fl*^*Mlkl*^*fl/fl*^ control mice. Megakaryocytes are indicated by asterisks. **d** Platelet counts at 2 h and 6 h post I.P. administration of LPS at 5 mg/ml. Mice were analysed in cohorts and blood was collected by cardiac puncture. 2 h LPS; Floxed control (WT) *n* = 3, *Caspase-8*^*Pf4Δ/Pf4Δ*^ *n* = 4, *Mlkl*^*Pf4Δ/Pf4Δ*^ *n* = 4 and *Caspase-8*^*Pf4Δ/Pf4Δ*^ *Mlkl*^*Pf4Δ/Pf4Δ*^ *n* = 3. 6 h LPS; WT *n* = 4, *Caspase-8*^*Pf4Δ/Pf4Δ*^ *n* = 4, *Mlkl*^*Pf4Δ/Pf4Δ*^ *n* = 6, and *Caspase-8*^*Pf4Δ/Pf4Δ*^ *Mlkl*^*Pf4Δ/Pf4Δ*^ *n* = 4. Platelet counts from 1 representative experiment. The experiment has been repeated 3 times. Statistically-significant differences are indicated relative to prebleed of WT, *Caspase-8*^*Pf4Δ/Pf4Δ*^*, Mlkl*^*Pf4Δ/Pf4Δ*^ *and Caspase-8*^*Pf4Δ/Pf4Δ*^ *Mlkl*^*Pf4Δ/Pf4*^ mice or where indicated. **e** Megakaryocyte numbers per FOV in H&E-stained sections of BM from LPS treated wild-type, *Caspase-8*^*Pf4Δ/Pf4Δ*^*, Mlkl*^*Pf4Δ/Pf4Δ*^ and *Caspase-8*^*Pf4Δ/Pf4Δ*^ *Mlkl*^*Pf4Δ/Pf4Δ*^ mice. 2 h LPS WT *n* = 5, *Caspase-8*^*Pf4Δ/Pf4Δ*^ *n* = 5, *Mlkl*^*Pf4Δ/Pf4Δ*^ *n* = 4 and *Caspase-8*^*Pf4Δ/Pf4Δ*^ *Mlkl*^*Pf4Δ/Pf4Δ*^ *n* = 4. 6 h LPS; WT *n* = 5, *Caspase-8*^*Pf4Δ/Pf4Δ*^ *n* = 5, *Mlkl*^*Pf4Δ/Pf4Δ*^ *n* = 6, and *Caspase-8*^*Pf4Δ/Pf4Δ*^ *Mlkl*^*Pf4Δ/Pf4Δ*^ *n* = 4. Data are presented as mean ± s.d, Two-way ANOVA relative to untreated for WT, *Caspase-8*^*Pf4Δ/Pf4Δ*^, *Mlkl*^*Pf4Δ/Pf4Δ*^ and *Caspase-8*^*Pf4Δ/Pf4Δ*^ *Mlkl*^*Pf4Δ/Pf4Δ*^ or where indicated with the Bonferroni multiple comparison test, *P < 0.05; **P < 0.005; ***P < 0.0005, ****P < 0.0001.

To ascertain the effect of LPS-induced necroptosis on platelet production and consumption, we conditionally deleted Caspase-8 and MLKL in the megakaryocytic lineage in vivo. For this, *Caspase-8*^*Pf4Δ/Pf4Δ*^ mice^18^ were crossed to *Mlkl*^*Pf4Δ/Pf4Δ*^ mice to generate *Caspase-8*^*Pf4Δ/Pf4Δ*^*Mlkl*^*Pf4Δ/Pf4Δ*^ (double KO or “DKO”) mice, with loss of Caspase-8 and MLKL proteins in purified platelet and megakaryocyte lysates confirmed via western blot (Fig. 7a). As expected, *Mlkl*^*Pf4Δ/Pf4Δ*^ and *Caspase-8*^*Pf4Δ/Pf4Δ*^*Mlkl*^*Pf4Δ/Pf4Δ*^ megakaryocytes were resistant to TSI-induced necroptosis (Supplementary Fig. 6), demonstrating efficient conditional deletion of *Mlkl*. Although *Caspase-8*^−*/−*^*Mlkl*^*−/−*^ mice rapidly develop severe lymphadenopathy, systemic autoimmune disease, and thrombocytopenia^41^, *Caspase-8*^*Pf4Δ/Pf4Δ*^*Mlkl*^*Pf4Δ/Pf4Δ*^ DKO mice were viable and fertile and did not exhibit any changes in platelet counts or other peripheral blood counts compared to wild-type floxed littermate controls (Table 1). Megakaryocytes were enumerated in H&E stained sternal sections from DKO and floxed control mice and their numbers and morphology were normal (Fig. 7b, c). TLR4 has previously been reported to be expressed on the surface of murine platelets and CD41^+^ megakaryocytes derived from foetal livers^54^, and we had already observed that it was expressed on BM-derived megakaryocytes (Fig. 2d). Next, to examine the role of LPS-mediated necroptosis in the megakaryocytic lineage in vivo, we challenged *Caspase-8*^*Pf4Δ/Pf4Δ*^*, Mlkl*^*Pf4Δ/Pf4Δ*^*, Caspase-8*^*Pf4Δ/Pf4Δ*^*Mlkl*^*Pf4Δ/Pf4Δ*^ DKO and wild-type control mice with 5 mg/ml LPS intraperitoneally for 2 h and 6 h. The body temperatures of mice from all genotypes declined similarly over time (Supplementary Fig. 7a). While RBC counts were not affected by LPS treatment at the timepoints assessed, mice from all genotypes became leukopenic at 2 h (Supplementary Fig. 7b, c). In response to LPS, wild-type mice became thrombocytopenic within 2 h, exhibiting significantly attenuated platelet counts that, while still reduced at 6 h, had started to rebound (Fig. 7d). Strikingly, *Caspase-8*^*Pf4Δ/Pf4Δ*^ and *Caspase-8*^*Pf4Δ/Pf4Δ*^*Mlkl*^*Pf4Δ/Pf4Δ*^ DKO animals were transiently protected from thrombocytopenia at 2 h, while *Mlkl*^*Pf4Δ/Pf4Δ*^ mice performed similarly to wild-type (Fig. 7d). We did not observe significant differences between the genotypes in sternal megakaryocyte numbers enumerated from H&E sections of LPS treated mice (Fig. 7e, Supplementary Fig. 7d) and numbers were similar to untreated mice (Figs. 5g, 7b). These findings imply that LPS-induced thrombocytopenia was due to increased platelet consumption by incorporation in blood clots and/or clearance rather than directly affecting megakaryocyte numbers and platelet production. Overall, these data implicate *Caspase-8*, but not *Mlkl*, deletion in the megakaryocytic lineage in transient protection from LPS-induced thrombocytopenia.

## Discussion

Our current study demonstrated for the first time that megakaryocytes can undergo necroptotic cell death. In vitro, we demonstrated that wild-type BM-derived megakaryocytes can undergo necroptosis following TNF stimulation (triggered by TSI), as assessed by loss of cell viability and phosphorylation of MLKL, which is a hallmark of pathway activation. Megakaryocytes displayed distinct features of necroptosis, including cell swelling and loss of membrane integrity when visualised by live cell time lapse video microscopy. As expected, based on studies of other cell types lacking MLKL, *Mlkl*^*−/*−^ and *Mlkl*^*Pf4Δ/Pf4Δ*^ megakaryocytes were resistant to TSI induced necroptosis. Live cell time lapse video microscopy revealed that necroptosis occurred rapidly in megakaryocytes, as early as 1 h post TSI treatment, while TS induced apoptosis was slower and evident by 4 h. Distinct from their progenitors, platelets did not express TNFR1 at marked levels, consistent with previous reports^36^, and therefore were largely refractory to TSI induced necroptosis in vitro.

Previously, it was reported that disabling necroptosis signalling with deletion of *Mlkl* in vivo had no effect on platelet counts and megakaryocyte numbers in BM and spleen at steady state^25,41^. We interrogated this further and found, in support of these findings, that the percentage of megakaryocytes and their ploidy profiles were normal in *Mlkl*^*−/−*^ BM. Furthermore, the recovery from antibody-mediated thrombocytopenia in *Mlkl*^−*/−*^ mice was similar to that in wild-type control mice, indicating that stress-induced platelet production was unaffected by loss of *Mlkl*. We further generated mice with a conditional deletion of *Mlkl* in the megakaryocytic lineage and demonstrated that platelet count and other peripheral blood counts in *Mlkl*^*Pf4Δ/Pf4Δ*^, mice were comparable to littermate controls, with no change in megakaryocyte numbers or morphology compared to floxed control mice. We conclude that, while megakaryocytes contain a functional necroptosis pathway, it is not required for steady state platelet production or recovery from antibody-mediated thrombocytopenia.

Recently, a role of necroptosis signalling proteins in thrombus formation was suggested based on the implication of RIPK3 in modulating in vivo thrombosis and haemostasis^32^. Defects in thrombin and thromboxane A2 induced platelet aggregation were evident in *Ripk3*^−*/*−^ platelets, linked to reduced dense granule, but not α-granule, release. *Ripk3*^*−/−*^ mice were reported to exhibit prolonged tail bleeding times and occlusion times in an in vivo model of arteriole thrombosis compared with their wild-type counterparts^32^. Another study showed that targeting necroptosis signalling with the specific RIPK1 antagonist Necrostatin-1 or genetic deletion of *Mlkl* systemically in vivo, partially protected mice from inferior vena cava ligation-induced venous thrombosis^62^. While loss of *Mlkl* attenuated thrombus size, mechanistically this was proposed to mainly be due to reduced neutrophil necroptosis and NET formation, however, this was not formally proven by conditional deletion of *Mlkl* in neutrophils. In our *Mlkl*^*−/−*^ mouse model, we report mild, but statistically significant, prolonged tail re-bleeding times compared to wild-type mice, indicating unstable thrombus formation. Therefore, our data support a role for MLKL in thrombus formation in vivo. RIPK3 is also known to function in pathways other than necroptosis, such as apoptosis and inflammatory cytokine signalling^41,63–65^. Therefore, a role of MLKL in thrombus formation is definitive evidence that necroptosis is involved in thrombus formation. We evaluated α-granule degranulation by P-selectin exposure and integrin αIIbβ3 activation in response to agonist-induced platelet activation by thrombin in wild-type, *Mlkl*^*−/−*^ and *Mlkl*^*Pf4Δ/Pf4Δ*^ platelets. We found a significant reduction in integrin activation in *Mlkl*^−/−^ and *Mlkl*^*Pf4Δ/Pf4Δ*^ platelets in response to thrombin, and degranulation assessed by P-selectin exposure. However, no difference between genotypes was evident in response to triggering GPVI (by convulxin) or PAR4 (by PAR4-activating peptide, AP) receptors. Thrombin is a key enzyme that functions in the coagulation cascade and a potent activator of platelets. In mice, thrombin activates platelets via N‐terminal cleavage of protease‐activated receptors (PARs) 3 and 4^66,67^, and *PAR4*^−*/−*^ mice have been shown to be non-responsive to thrombin induced activation^68^. Our data suggested a defect in thrombin signalling independent of PAR4 signalling, which is a surprising finding. Thrombin and PAR4-AP activate the PAR4 receptor by different mechanisms. Thrombin binds exosites leading to enzymatic cleavage and activation, while PAR4-AP binds to active sites directly. Therefore, we speculate that the exosites in PAR4 are less available on platelets in *Mlkl*^*−/*−^ and *Mlkl*^*Pf4Δ/Pf4Δ*^ mice causing the low dose thrombin defect. Similar to the *Ripk3*^*−/*−^ platelet study^32^, we report reduced platelet activation and aggregation in response to low dose thrombin. However, we observed both reduced integrin conformational change and α-granule release, in contrast to the *Ripk3*^*−/*−^ platelet study, which reported normal α-granule release. Further analyses of the role of MLKL in platelet function revealed that loss of MLKL did not improve the PSL, although this will need to be confirmed in a more comprehensive study involving additional markers. In addition, loss of MLKL did not play a measurable role in the generation of procoagulant platelets in vitro.

Megakaryocytes have been reported to undergo cell death when producing platelets^69^. However, recent studies have shown that while the intrinsic and extrinsic apoptosis cell death pathways are functional in megakaryocytes^69,70^, they need to be restrained in order to produce platelets^14,18^. It was formally possible that other types of cell death, such as necroptosis could play an active role during in vivo platelet formation, in particular in a setting of inflammation or in response to acute needs. In the current study, we employed live cell video microscopy to not only assess megakaryocyte viability in response to stimulation of necroptosis signalling, but to ascertain whether megakaryocytes release platelets upon undergoing necroptosis. From our imaging experiments, we were unable to detect platelet formation or release in response to TSI treatments of wild-type megakaryocytes when images were acquired every 10 min for a 7 h period. This suggests that activation of necroptosis does not stimulate platelet production in vitro, although it can trigger megakaryocyte rupture. One caveat of our study is that platelet release might occur too quickly for us to capture it, because unfortunately, when we attempted faster imaging, megakaryocyte viability was negatively affected. Our in vitro finding that megakaryocyte necroptosis triggers megakaryocytic death is supported by in vivo evidence that *Mlkl*^*D139V/D139V*^ mice, which harbour a constitutively-activated form of MLKL, displayed attenuated liver megakaryocyte numbers at P2 and were shown to be thrombocytopenic at P3^31^. Moreover, adult mice heterozygous for *Mlkl*^*D139V*^ had impaired platelet recovery from chemotherapy and irradiation treatment^31^. It is therefore likely that megakaryocytes must restrain necroptosis to survive and synthesise platelets. This finding may have important implications for our understanding of human diseases where cell death of megakaryocytes has been implicated and it is tempting to speculate that the pathway could be activated by cytotoxic drugs, autoantibodies, viral or bacterial infections.

We report that BM-derived megakaryocytes and blood platelets both express TLR4 on their surface, as assessed via flow cytometry, prompting us to assess the effect of loss of Caspase-8 and MLKL on platelet production and clearance in an in vivo LPS-induced sepsis model. It is unclear as to how LPS induces thrombocytopenia, however, it has been shown that administration of LPS in vivo can promote platelet aggregate formation in the lung and liver microvascular circulation, resulting in profound thrombocytopenia in wild-type mice^55,71^. Our data support a model where LPS promotes platelet consumption/clearance, rather than directly affecting megakaryocytes. While we observed rapid thrombocytopenia at 2 and 6 h post LPS administration, megakaryocyte numbers remained unperturbed. Interestingly, we found a transient protection to thrombocytopenia in *Caspase-8*^*Pf4Δ/Pf4Δ*^ and *Caspase-8*^*Pf4Δ/Pf4Δ*^ *Mlkl*^*Pf4Δ/Pf4Δ*^ (DKO) mice, but not *Mlkl*^*Pf4Δ/Pf4Δ*^ mice. Conditional deletion of *Caspase-8* in megakaryocytes and platelets, could either protect from extrinsic apoptosis or sensitize the megakaryocytic lineage to necroptosis. Since the process was independent of MLKL, the data indicate that platelets unable to undergo extrinsic apoptosis were transiently protected. This finding is also in line with previous reports that Caspase-8 is activated during sepsis triggered by LPS^72,73^. We have previously not been successful in triggering extrinsic apoptosis in platelets by FasL^18^, however, this study suggests that the extrinsic apoptosis pathway is functional in platelets in response to other stimuli, such as LPS. While we did not implicate LPS-induced thrombocytopenia in the activation of necroptosis in megakaryocytes and platelets, our study provides novel mechanistic insight into a role of activation of extrinsic apoptosis in platelets in LPS-induced thrombocytopenia and sepsis. Pathogenic bacteria isolated from patients with sepsis and M1 protein from *Streptococcus pyogenes* have been shown to trigger apoptosis of human platelets in vitro^74,75^, suggesting a mechanism by which bacterial pathogens might cause thrombocytopenia in patients with blood stream infections. Future studies are needed to investigate if the mechanistic insight gained from our in vivo study could help the development of targeted therapies combating thrombocytopenia in sepsis patients.

In summary, we provide evidence of a functional necroptosis cell death pathway in megakaryocytes. Megakaryocytes stimulated to undergo TNF-directed necroptosis lost viability and displayed cell swelling and loss of membrane integrity. Analyses at steady state and post antibody-mediated thrombocytopenia revealed that platelet production was normal in the absence of MLKL, however low dose thrombin-mediated platelet activation and haemostasis were impaired. While necroptosis needs to be restrained in order to keep megakaryocytes alive, it is not required for platelet production.

## Materials and methods

### Mice

*Mlkl*^*-/-*^^25^, *Caspase-8*^*Pf4∆/Pf4∆*^^18^, *Pf4-Cre*^76^, and *Mlkl*^*D139V*^ mice^31^ have been previously described. MLKL conditional knockout mice were generated according to^25^. Mice carrying a Targeted *Mlkl* locus with exon 3 flanked by loxP sites and including a neo-resistance cassette were crossed with transgenic mice ubiquitously expressing Flp recombinase^77^ to remove the neo cassette, resulting in mice with the conditional MLKL locus. These mice were crossed with floxed Caspase-8, PF4 Cre-expressing mice*, Caspase-8*^*Pf4∆/Pf4∆*^^18^ to generate Caspase-8 and MLKL conditional deletion in megakaryocytes and platelets, *Caspase-8*^*Pf4∆/Pf4∆*^*Mlkl*^*Pf4∆/Pf4∆*^. All mutations had been backcrossed onto the C57BL/6 background for at least 10 generations prior to this study. Mice were 7–12 weeks old and experiments include balanced groups of male and female mice if not otherwise stated.

### Materials

DMSO (D2650), Thiazole orange (390062), ADP (A2754), ATP (A1825), Thrombin (T9326-150UN), PAR4-AP (A3227-1 MG) and Fibrinogen (F3879) were from Sigma-Aldrich (St. Louis, MO USA). Convulxin (ALX-350-100-C050) and QVD-OPH were purchased from Enzo Life Sciences (Farmingdale, NY USA) and tetramethylrhodamine, methyl ester (TMRM) from Thermo Fisher Scientific (Waltham, MA USA). Calibration beads 3.5–4.0 μm were from Spherotech Inc (Lake Forest, IL USA). Fluorescently conjugated antibodies used for flow cytometry included: JON/A (Integrin αIIbβ3, M023-2), GPIX (CD42a, M051-1), GPIbα (CD42b, M040-3), and GPVI (JAQ1, M011-1) from Emfret Analytics GmbH and Co. KG (Eibelstadt, Germany); fluorescently conjugated anti–mouse, CD41 (MWReg30), anti-P-selectin (CD62P) RB40.34 (561923, BD Biosciences) (Franklin Lakes NY, USA) and Annexin V (A13199), Life Technologies. Anti-platelet serum was purchased from Cedarlane (Burlington, Ontario, Canada). TNF (Fc-hTNF was produced in house), Necrostatin-1 (Sigma-Aldrich), the SMAC Mimetic, Compound A^38^ and IDN-6556 (pan caspase inhibitor) were gifts from TetraLogic (Malvern, PA, USA). Antibodies: TLR-4-PE (ebiosciences), Mlkl (Clone 3H1, in-house WEHI Monoclonal Facility, Rat 1:2000; available as Millipore MABC604), RIPK1 (BD Biosciences, mouse 1:1000), RIPK3 (ProSci, rat 1:1000), TNFR1 (MAB430-SP, R&D systems), anti-TNFR1 antibody (Abcam ab223352), Direct-blot HRP anti-GAPDH (607903, Biolegend), anti-Pro Caspase-8 (#4927, Cell Signaling Technology) anti-Phospho (pS358) MLKL (ab196436 Abcam), anti-Phospho RIPK1 (ab195117 Abcam), anti-Phospho RIPK3 (#65746, Cell Signaling Technology), anti-β-Actin (Sigma-Aldrich), anti-vWF (Polyclonal rabbit A0082; Dako), ABC kit (Vector laboratories PK-6100), DAB solution (Vector Laboratories) and other reagents ABT-737 (WEHI in-house), StemPro^TM^-34 SFM (ThermoFisher Scientific), HRP-conjugated secondary antibodies (Santa Cruz Biotechnology Inc.).

### Haematology

Automated cell counts were performed on blood collected from the retro-orbital plexus, into Microtainer tubes containing EDTA (Sarstedt, Ingle Farm, SA, Australia), using an Advia 2120 hematological analyzer (Siemens, Munich Germany). Organs were collected in 10% formalin or 4% PFA and H&E sections prepared for pathological analysis. Megakaryocytes were counted manually in a blinded fashion in sections of sternum and stained with H&E with a minimum of 10 high power fields (200×) analysed.

### Platelet preparation

Murine blood was obtained by cardiac puncture into 0.1 volume of Aster-Jandl anticoagulant (85 mM sodium citrate, 69 mM citric acid, and 20 mg/ml glucose, pH 4.6)^78^. Platelet-rich plasma (PRP) was obtained by centrifugation at 125 g for 8 min, followed by centrifugation of the supernatant buffy coat at 125 g for 8 min. Platelets were washed by two sequential centrifugations at 860 × *g* for 5 min in 140 mM NaCl, 5 mM KCl, 12 mM trisodium citrate, 10 mM glucose, and 12.5 mM sucrose, pH 6.0 (buffer A). The platelet pellet was resuspended in 10 mM Hepes, 140 mM NaCl, 3 mM KCl, 0.5 mM MgCl_2_, 10 mM glucose, and 0.5 mM NaHCO_3_, pH 7.4 (buffer B) at a concentration of 1–5 × 10^8^ cells/ml. The platelet count was determined by flow cytometric analysis by adding a known concentration of FACS calibration beads (Spherotech).

### Primary megakaryocyte culture

Murine bones (2× femura and 2x tibiae) were removed and bone marrow was flushed in KDS-BSS with 2% FCS. The cells were lineage depleted by incubation with a mix of biotinylated antibodies (CD4, CD2, CD3, CD5, CD8, Ter119, B220, CD19, Gr-1, Ly6G, F4/F8, CD127; WEHI) in KDS-BSS 2% FCS, followed by anti-biotin magnetic microbeads (Miltenyi Biotec, NSW, Australia) and MAC LS columns (Miltenyi Biotec). Single cell suspensions were cultured for 3–5 days at 5 × 10^5^ cells per ml in serum-free medium (StemPro^TM^-34 SFM), supplemented with 100 ng/ml murine TPO (Peprotech) at 37 °C, 5% CO_2_ and mature megakaryocytes purified using a discontinuous BSA density gradient (3, 1.5 and 0%). Cells were harvested in the 3% layer after 35 min.

### SDS PAGE and western blot analysis

Washed BSA gradient-purified megakaryocytes (3 × 10^4^ cells per ml in SFM with TPO) were seeded into 24 well plates and then incubated at 37 °C, 5% CO_2_ with or without the addition of TNF (100 ng/ml), Smac mimetic (compound A; 500 nM), IDN-6556 (pan caspase inhibitor; 5 µM), Necrostatin-1 (50 µM) for 2 h and 4 h time points. Purified platelets were lysed in NP40 lysis buffer and untreated or treated megakaryocytes were harvested and lysed in RIPA buffer with phosphatase and Complete protease inhibitors (Roche). Proteins were separated on 4–12% Bis-Tris NuPAGE protein gels (Invitrogen) under reducing conditions, transferred onto Immobilon-P membrane and immunoblotted with RIPK1, RIPK3, MLKL, Phosho-MLKL, TNFR1, β-actin and GAPDH antibodies, followed by incubation with secondary HRP-conjugated antibody and enhanced chemiluminescence.

### Immunohistochemistry

For vWF staining on P2 neonatal livers collected in 4% PFA, Sagittal sections were dewaxed and antigen retrieved by heating in citrate buffer, pH 6.0. Endogenous peroxide activity was blocked with H_2_O_2_ and slides were preincubated with 10 % FCS, 1% BSA in PBS blocking buffer. Sections were incubated with vWF polyclonal antibody followed by biotinylated secondary antibody. ABC vectastain was used prior to revealing the staining with DAB solution. Stained sections were counterstained with haematoxylin and slides were mounted to visualize under the microscope. vWF-positive megakaryocytes were counted manually with a minimum of 33 high power fields (200×) analysed.

### Cell viability assays

BSA gradient-purified megakaryocytes (3 × 10^4^ cells per ml in SFM with TPO) were seeded into 96-well plates and purified platelets (1 × 10^8^ platelets per well in buffer B) from wild type mice were seeded in a 96 well round bottom plate and then incubated at 37 °C, 5% CO_2_ with or without the addition of TNF (100 ng/ml), Smac mimetic (compound A 500 nM), IDN-6556 (pan caspase inhibitor 5 µM), Necrostatin-1 (50 µM). Cell titre glo ATP reagent (Promega) was added to megakaryocytes 4.5 h and 7 h after treatment and to platelets after 1.5 h and 3 h to determine cell viability by measuring ATP levels. The luminescence of each sample was determined in a LumiSTAR Galaxy luminometer (BMG Labtech).

### Live cell imaging

BSA gradient-purified megakaryocytes (500 cells per 400 µl in SFM supplemented with TPO) were seeded into Ibidi 8 well chamber slides (Ibidi) and (400 cells per 200 µl) in optical black bottom 96 well plates (Nunc) with or without the addition of TNF (100 ng/ml), Smac mimetic (compound A 500 nM), IDN-6556 (pan caspase inhibitor 5 µM), Necrostatin-1 (50 µM). Treated megakaryocytes were subjected to time lapse microscopy on Zeiss live cell Axio inverted modular microscope and images were acquired every 10 min for a 7 h period. For quantification of propidium iodide fluorescence intensity per well, a custom macro in Fiji software was applied as previously described^79^ using an intensity threshold, followed by object splitting and size filtering steps.

### Platelet flow cytometry

To determine the surface expression of platelet receptors, purified platelets were stained with fluorescently conjugated antibodies to platelet Integrin-αIIb (CD41), GPIbα (CD42b), GPIX (CD42a), GPVI for 20 min in buffer B at room temperature and samples were further diluted and directly examined by flow cytometry (Cytoflex, BD). In platelet activation experiments, purified platelets were treated with or without the single agonists ADP (12.5–50 µM), Convulxin (12.5–50 ng/ml), PAR4-AP (0.125–0.2 mM) and thrombin (0.0625–0.25 U/ml) for 20 min at room temperature (37 °C for ADP) in the presence of 1 mM CaCl_2_ and activation of the αIIbβ3 integrin (JON/A) or P-selectin exposure were assessed by flow cytometry.

### TLR4 surface expression

Purified platelets and 5-days cultured BM derived megakaryocytes (see primary megakaryocyte culture) were incubated with CD41-APC and TLR4-PE Abs for 30 min, washed with KDS-BSS + 5% FCS, and data was acquired on Cytoflex and LSRII (BD), respectively. CD41 and TLR4 expression were analysed on FlowJo software. Megakaryocytes were not gradient purified.

### Stressed platelet production

Acute thrombocytopenia was induced by I.V. injection of 100 μl of APS (Cedarlane) per 20 g body weight at a 1:25 pre-dilution. This reduces platelet counts to near undetectable levels within 24 h, followed by rebound thrombocytosis^40^. Platelet counts were assessed at 72 h and 120 h post APS using an Advia 2120 hematological analyser. Reticulated (young) platelets were enumerated by thiazole orange staining. Thiazole orange powder was dissolved in methanol at 1 mg/ml then pre-diluted at 1:10,000 in PBS. To label platelets, CD41-PE was diluted 1:30 in PBS. Samples were prepared by mixing 7 µl of blood (3 μl of tail vein blood was collected in a Microtainer tubes containing EDTA mixed with 14 μl of buffer B and 3 µl of anti-coagulant) with 10 µl of CD41-PE and 50 µl of diluted thiazole in PBS. Samples were incubated for 20 min at RT, fixed with 1% PFA in PBS and analysed on a Cytoflex.

### Megakaryocyte ploidy

Bone marrow was flushed from murine femura and tibiae into 1 ml CATCH media (3% BSA, 3% FBS, 0.38%w/v Tri-sodium citrate, 1 mM Adenosine, 2 mM Theophylline in HBSS) on ice. The sample was stained with 4 μg/ml CD41-APC antibody and incubated on ice for 25 min. Unbound antibody was washed off in CATCH buffer by centrifuging at 500 rpm for 5 min at 4 °C. Supernatant was removed until 200 μl remained and cells were resuspended in 3 ml of propidium iodide (PI) 0.05 mg/ml PI in 0.1% Tri sodium citrate solution and incubated on ice for 35 min. Stained cells were washed and 5 μl RNase (50 µg/ml) was added and the sample was incubated at room temperature in the dark for 30 min. CD41^+^ events were collected and analysed on BD LSRII flow cytometer. Analysis was performed using FlowJo software (BD Biosciences, version 10.0.7).

### Bleeding time

Haemostasis was assessed, as described previously^80^, by 3-mm tail amputation. The mouse tail was transected at 3 mm from the tip and immediately immersed into 37 °C saline. The bleeding time was determined as the time from the tail transection to the moment the blood flow stopped for more than 2 min. Bleeding time beyond 10 min was considered as the cut-off time for the purpose of statistical analysis. RBCs were pelleted and lysed in 1 ml H_2_O. Haemoglobin was quantified by absorbance at 570 or 575 nm (Beckman DU®530 Life Science UV/Vis Spectrophotometer). The investigator was blinded to the genotype during the experiment. Analysis of re-bleeding events: Following cessation of bleeding for 2 min, the incision was monitored for a further 2 min, and further bleeding within this period classified as ‘re-bleeding’. The duration of re-bleeding was monitored as described above. Haemostasis was measured in anesthetized mice (ketamine 100 mg/kg; xylazine 20 mg/kg I.P.).

### LPS-induced thrombocytopenia

*Caspase-8*^*Pf4∆/Pf4∆*^, *Mlkl*^*Pf4∆/Pf4∆*^, *Caspase-8*^*Pf4∆/Pf4∆*^*Mlkl*^*Pf4∆/Pf4∆*^ and floxed littermate control mice between 7–10 weeks of age were injected intraperitoneally with a single dose of ultra pure LPS (5 mg/ml) from *E. coli*. Mice were divided into two cohorts; 2 h or 6 h LPS and rectal body temperatures were monitored to assess animal health. Mice were analysed at 2 h or 6 h post LPS administration. Blood was drawn by cardiac puncture and collected into microtainer tubes containing EDTA for peripheral blood counts. The sternum was collected in 4% PFA for H&E sections to be prepared.

### Statistical analyses

Statistical significance between two treatment groups was analysed using an unpaired Student’s *t* test with two tailed *P* values. One-way ANOVA or Two-way ANOVA with multiple comparison test was applied where appropriate (GraphPad Prism Software Version 8). *P < 0.05; **P < 0.005; ***P < 0.001, ****P < 0.0001 or as otherwise stated. The variance was similar between statistically compared experimental groups. Either one-way or two-way analysis of variance (ANOVA) was used based on the experimental design. Data are presented as mean ± SD or SEM.

## Supplementary information

Supplementary information

## Data Availability

All data are available on request from the authors. Figure 3d has a Supplementary figure with raw data associated with it; refer to Supplementary Fig. 2c.
