# Supplementary material for: The necroptotic cell death pathway operates in megakaryocytes, but not in platelet synthesis
Source: Cell Death Dis. 2021 Jan 28;12(1):133. doi: 10.1038/s41419-021-03418-z (PMC7843594; doi:10.1038/s41419-021-03418-z)
Supplement: Supplementary file 1 — Supplementary information [file 41419_2021_3418_MOESM1_ESM.pdf]

Supplementary Information

The necroptotic cell death pathway operates in megakaryocytes, but not in platelet synthesis

Moujalled and Gangatirkar et al., 2020

Supplementary methods

Procoagulant platelets.

Purified platelets were counted and adjusted to $1x10^8$ platelets/ml in buffer B and treated with a combination of (Thrombin 0.625-0.25 U/ml and Convulxin 25-50 ng/ml) for 10 minutes at room temperature. Treated platelets were stained with combination of Annexin-FITC, CD41-APC, and TMRM in buffer B containing 1mM Ca^{2+} and incubated at room temperature for 20 minutes. The procoagulant response was assessed by flow cytometry.

Platelet aggregation.

For aggregation studies, washed mouse platelets in modified Tyrode-buffer (10mM Hepes, 145 mM NaCl, 5 mM KCl, 2mM $MgCl_2$, 3.5 mM Na_2HPO_4, 10% Glucose and 10% BSA, pH 7.4) were stirred at 37°C in a four-channel automated platelet analyzer (AggRAM, Helena Laboratories, Tyne and Wear, UK) in the presence of 1 mM Ca^2 and 66.7 µg/ml fibrinogen. Platelets (80 µl with 5 $x10^8$ cells/ml), were mixed with 240 µl Tyrode-buffer buffer containing Ca^{2+} and fibrinogen. The extent of platelet aggregation 5 min post agonist stimulation was defined as the percentage change in optical density as measured by the automated platelet analyzer.

Platelet storage assay.

Purified platelets were adjusted to 1.5 x 10^8 platelets/ml in modified Tyrode-buffer containing 5% plasma and stored in 96 well round bottom plates in an incubator/orbital shaker I26 (New Brunswick Scientific Co Inc. Edison, NJ, USA) at 22°C with agitation at 60 rpm. The incubator was opened twice daily to allow the exchange of oxygen. At indicated timepoints, the platelet surface receptors GPIbα and GPVI were assessed by flow cytometry and cell viability (ATP) was measured by the Cell titer glo (Promega) assay.

Megakaryocytes treated with LPS and caspase inhibitor *in vitro*.

Megakaryocytes were cultured as described in materials and methods/primary megakaryocyte culture. BSA gradient-purified megakaryocytes ($3x10^4$ cells per ml in serum-free medium, StemPro^{TM}-34, with TPO) from wild type mice were seeded into 96-well plates and incubated at 37°C, 5% CO_2 with or without the addition of LPS (100 ng/ml or 1µg/ml) -/+ caspase inhibitors QVD-Oph (50 µM), IDN-6556 (5µM) or Zvad (10 µM), and Necrostatin-1 (50 µM) supplemented with or without 5% or 10% serum. Cell titer glo reagent (Promega) was added

to megakaryocytes after 20 and 22h of treatment to determine cell viability by measuring ATP levels. The luminescence of each sample was determined in a LumiSTAR Galaxy luminometer (BMG Labtech).

Supplementary figures

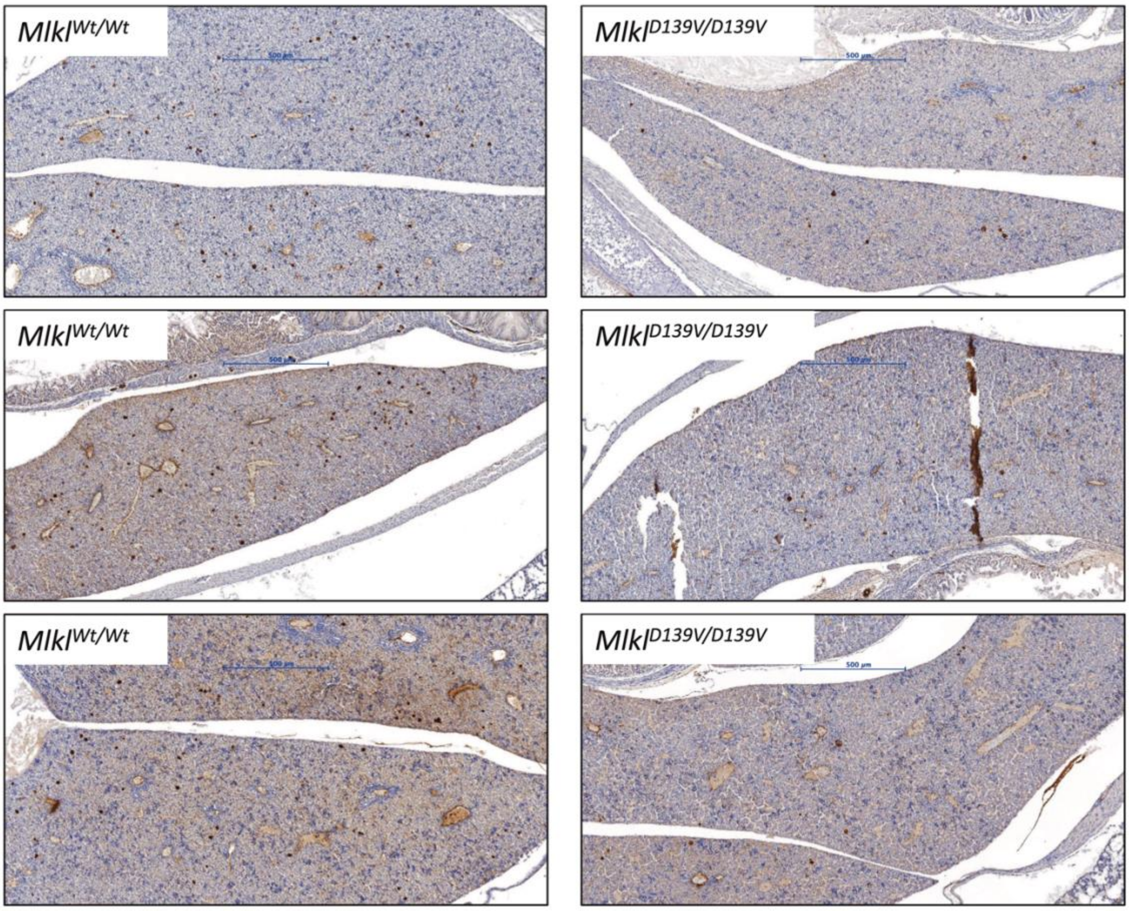

Supplementary figure 1: Liver megakaryocyte numbers are reduced in newborn *Mlkl*$^{D139V/D139V}$ mice.

Representative vWF staining (brown) of liver megakaryocytes from 3 *Mlkl*$^{Wt/Wt}$ and 3 *Mlkl*$^{D139V/D139V}$ P2 mice. Stained sections were counterstained with hematoxylin. The scale bars are 500µm.

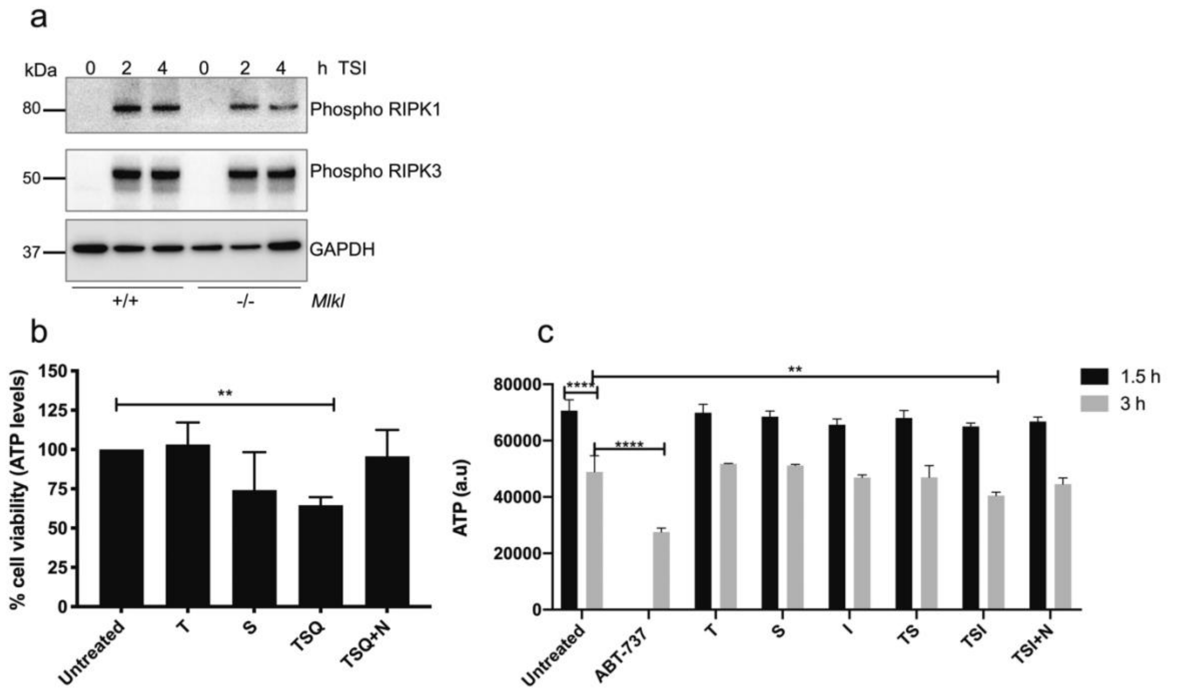

Supplementary figure 2: Megakaryocytes undergo necroptosis *in vitro*.

(a) Western blot analysis of protein lysates from WT or *Mlkl*$^{-/-}$ BM-derived cultured megakaryocytes untreated or treated with TSI (TNF; 100ng/ml, Smac mimetic; 0.5µM and IDN-6556; 5µM) for 2h and 4h at 37°C and probed for the indicated proteins. GAPDH was used as a loading control. **(b)** Cell viability in gradient purified cultured BM-derived megakaryocytes from WT mice untreated or treated with the indicated agents (TNF; 100ng/ml (T), Smac mimetic; 0.5µM (S), QVD-OPh; 50µM (Q), Necrostatin-1; 50 µM (N). ATP levels in megakaryocytes were measured 4.5 h after treatment at 37°C using the Cell Titer-Glo assay relative to untreated control. n=3 independent experiments and 3 biological replicates. Data represent mean ± s.d. One-way ANOVA with the Dunnett multiple comparison test. **(c)** Raw data values (ATP) in purified blood platelets from WT mice untreated or treated with the indicated agents (TNF; 100ng/ml (T), Smac mimetic; 0.5µM (S), IDN-6556; 5µM (I), Necrostatin-1; 50 µM (N). and 1 µM ABT-737). ATP levels in platelets were measured 1.5 and 3 h after treatment at 37°C using the Cell Titer-Glo assay, n=3 biological replicates, data represent mean ± s.d. Two-way ANOVA with the Dunnett multiple comparison test. ** P<0.005, ****P<0.0001.

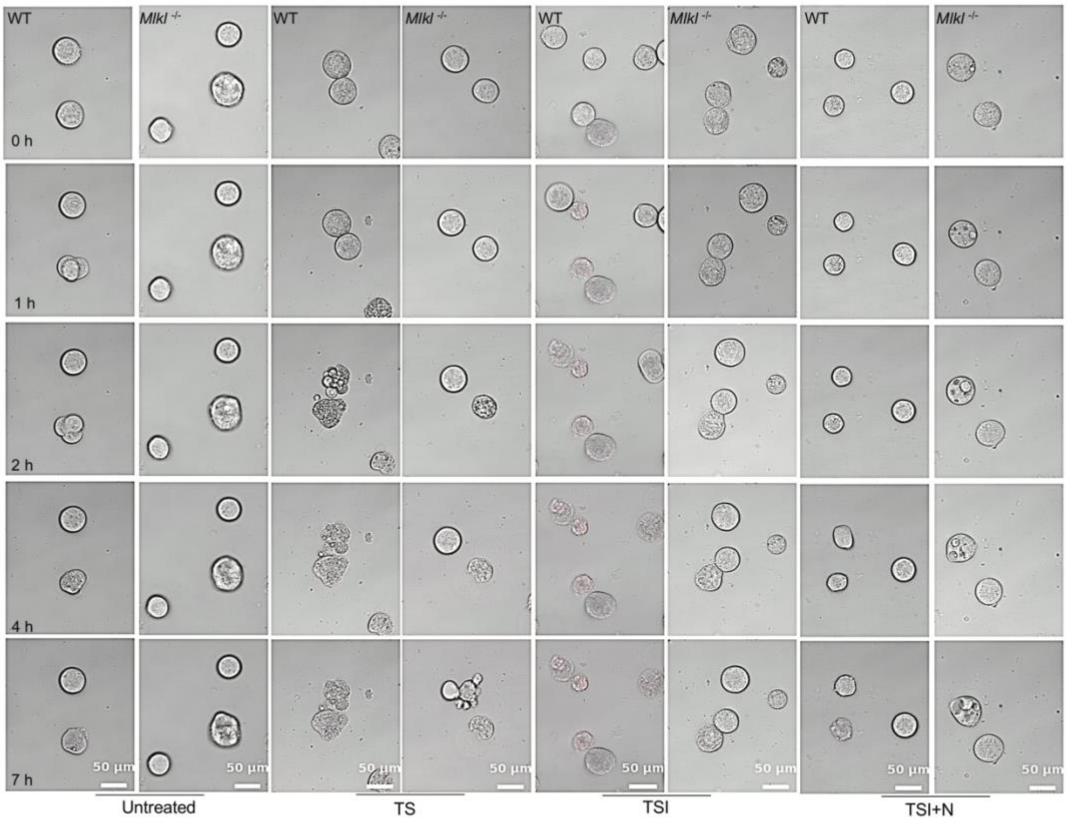

Supplementary figure 3: TNF induced necroptosis and apoptosis in megakaryocytes. Representative time lapse confocal images from WT and *Mlkl* $^{-/-}$ gradient purified cultured BM derived megakaryocytes treated with the indicated agents; TNF; 100ng/ml (T), Smac mimetic; 0.5µM (S), IDN-6556; 5µM (I), Necrostatin-1; 50µM (N) at the indicated time points (hours) at 37°C. PI is shown in red. Bar 50 µm. Cells were maintained on the stage within a humidified chamber at 37°C and 5% CO_2 for 7h. Images were acquired on a Ziess live cell Axio inverted modular microscope for live cell imaging every 10 minutes, driven by AxioVision v4.8 software. n=3 independent experiments.

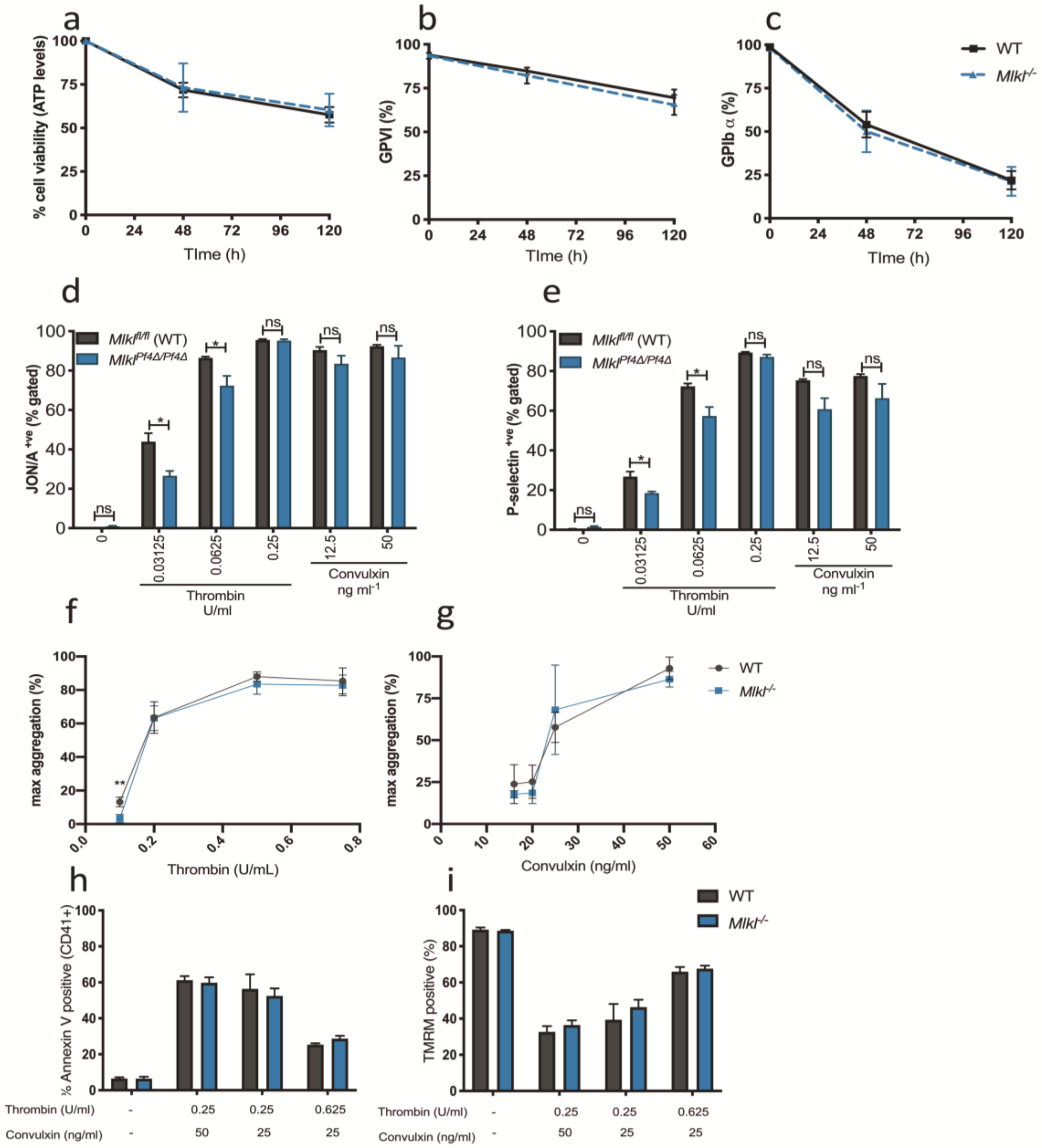

a
% cell viability (ATP levels)
TIme (h)
b
GPVI (%)
TIme (h)
c
GPIb α (%)
TIme (h)
WT
Mlkl-/-
d
JON/A +ve (% gated)
Mlklfl/fl (WT)
MlklPf4Δ/Pf4Δ
ns
*
Thrombin U/ml
Convulxin ng ml-1
e
P-selectin +ve (% gated)
Mlklfl/fl (WT)
MlklPf4Δ/Pf4Δ
Thrombin U/ml
Convulxin ng ml-1
f
max aggregation (%)
Thrombin (U/mL)
g
max aggregation (%)
Convulxin (ng/ml)
WT
Mlkl-/-
h
% Annexin V positive (CD41+)
Thrombin (U/ml)
Convulxin (ng/ml)
i
TMRM positive (%)
WT
Mlkl-/-
Thrombin (U/ml)
Convulxin (ng/ml)

Supplementary figure 4: Platelet function in the absence of MLKL.

(a, b, c) Murine platelets were stored at RT (22°C) with gentle agitation in 5% plasma for up to 5 days. **(a)** ATP levels in platelets determined by CellTiter-Glo assay. **(b)** GPVI and **(c)** GPIb α surface expression levels by flow cytometry n=3 mice per genotype. **(d, e)** Agonist-induced platelet activation in $Mlkl^{fl/fl}$ (WT) and $Mlkl^{Pf4\Delta/Pf4\Delta}$ platelets determined by **(d)** integrin activation (JON/A) or **(e)** P-selectin positive platelets by flow cytometry, statistically significant differences are shown relative to WT control platelets at the indicated concentration of agonist. Platelets were washed and counts adjusted before 20 minute incubations with Convulxin and Thrombin at the indicated concentrations. n=3 mice per genotype. **(f, g)** Maximal washed platelet aggregation in response to thrombin or convulxin n=3-6 mice per genotype. **(h, i)** Procoagulant response of WT and $Mlkl^{-/-}$ platelets in response to dual agonist treatment with thrombin and convulxin. Phosphatidylserine externalization measured by Annexin V binding in **h**. Platelet $\Delta\psi_m$ was assessed by flow cytometry using the cationic dye TMRM in **i**. TMRM retention within mitochondria is dependent on the maintenance of $\Delta\psi_m$, and loss of $\Delta\psi_m$ results in decreased TMRM fluorescence, n=3 mice per genotype. Data represent mean ± s.d. student's unpaired t-test, *P<0.05, ** P<0.005, ns; not significant.

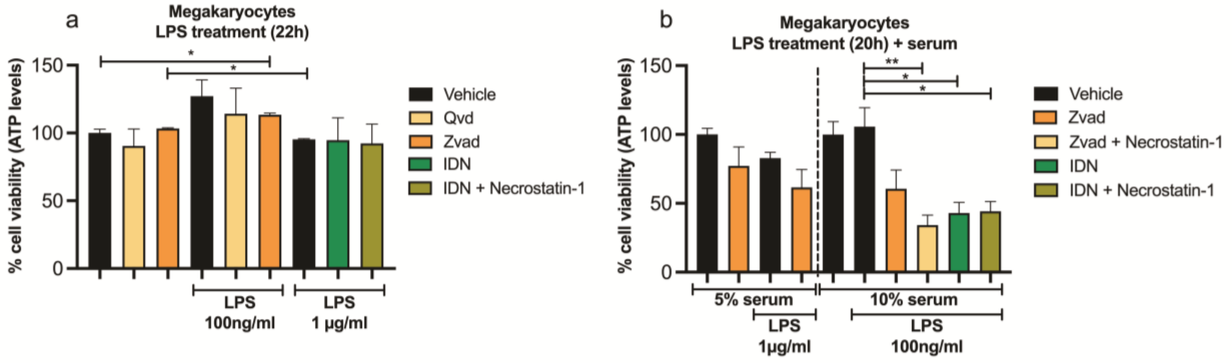

Supplemental Figure 5: Megakaryocytes treated with LPS and caspase inhibitor *in vitro*. Cell viability in BM-derived cultured megakaryocytes from WT mice untreated (vehicle) or treated with the indicated agents, LPS 100ng/ml or 1µg/ml; QVD-Oph 50µM; Zvad 10µM; IDN-6556; 5µM (IDN), Necrostatin-1; 50 µM. ATP levels in megakaryocytes were measured 22 h in (**a**) or 20 h in (**b**) after treatment at 37°C using the Cell Titer-Glo assay. (**a**) Data are presented as % relative to untreated vehicle control. Data combined from 4 independent experiments. n=2-8 replicates. Data represent mean ± SEM. One-way ANOVA with Dunnett's multiple comparison test. Significant differences are indicated. *$P<0.05$ (**b**) Data are presented as % relative to untreated vehicle control (5% or 10% serum). Data combined from 3 independent experiments. n=4 replicates (vehicle 10% serum, n=2). Data represent mean ± SEM. One-way ANOVA with Tukey's multiple comparison test. Significant differences are indicated. *P<0.05, **P<0.005.

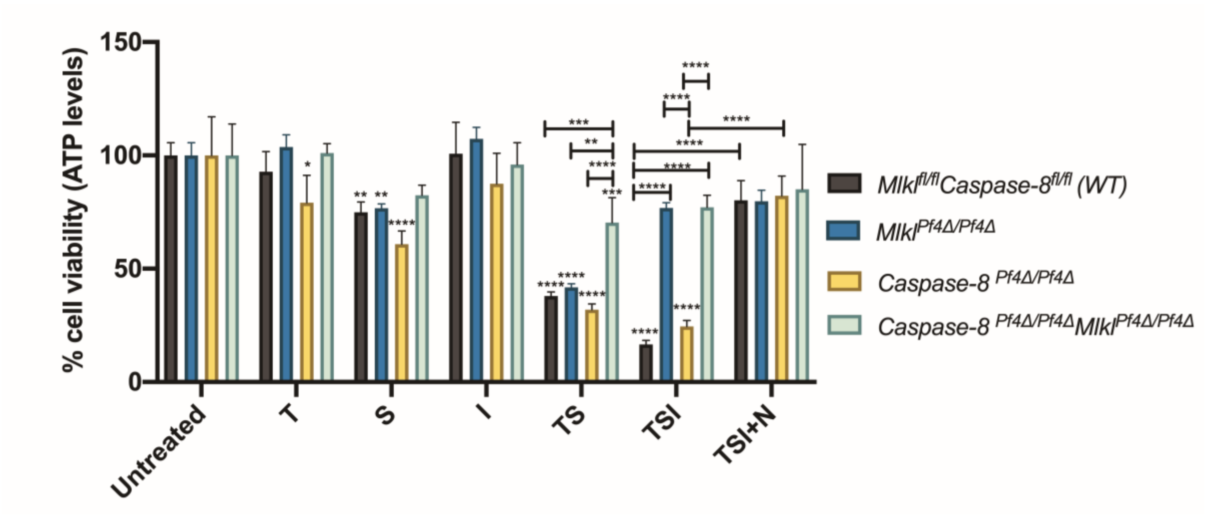

Supplementary figure 6: Cell viability in WT, *Mlkl*$^{Pf4\Delta/Pf4\Delta}$, *Caspase-8*$^{Pf4\Delta/Pf4\Delta}$ and *Mlkl*$^{Pf4\Delta/Pf4\Delta}$*Caspase-8*$^{Pf4\Delta/Pf4}$ BM-derived megakaryocytes in response to TS and TSI.

Cell viability in BM-derived cultured megakaryocytes from *Mlkl*$^{fl/fl}$*Caspase-8*$^{fl/fl}$ (WT), *Mlkl*$^{Pf4\Delta/Pf4\Delta}$, *Caspase-8*$^{Pf4\Delta/Pf4\Delta}$ and *Mlkl*$^{Pf4\Delta/Pf4\Delta}$*Caspase-8*$^{Pf4\Delta/Pf4}$ mice untreated or treated with the indicated agents (TNF; 100ng/ml (T), Smac mimetic; 0.5µM (S), IDN-6556; 5µM (I), Necrostatin-1; 50 µM (N). ATP levels in megakaryocytes were measured 4.5 h after treatment at 37°C using the Cell Titer-Glo assay relative to untreated control for each genotype, n=3 per genotype. Data represent mean ± s.d. Two-way ANOVA with the Dunnett multiple comparison test relative to untreated control WT, *Mlkl*$^{Pf4\Delta/Pf4\Delta}$, *Caspase-8*$^{Pf4\Delta/Pf4\Delta}$ and *Mlkl*$^{Pf4\Delta/Pf4\Delta}$*Caspase-8*$^{Pf4\Delta/Pf4}$, or where otherwise indicated. *P<0.05, **P<0.005, ***P<0.001, ****P<0.0001.

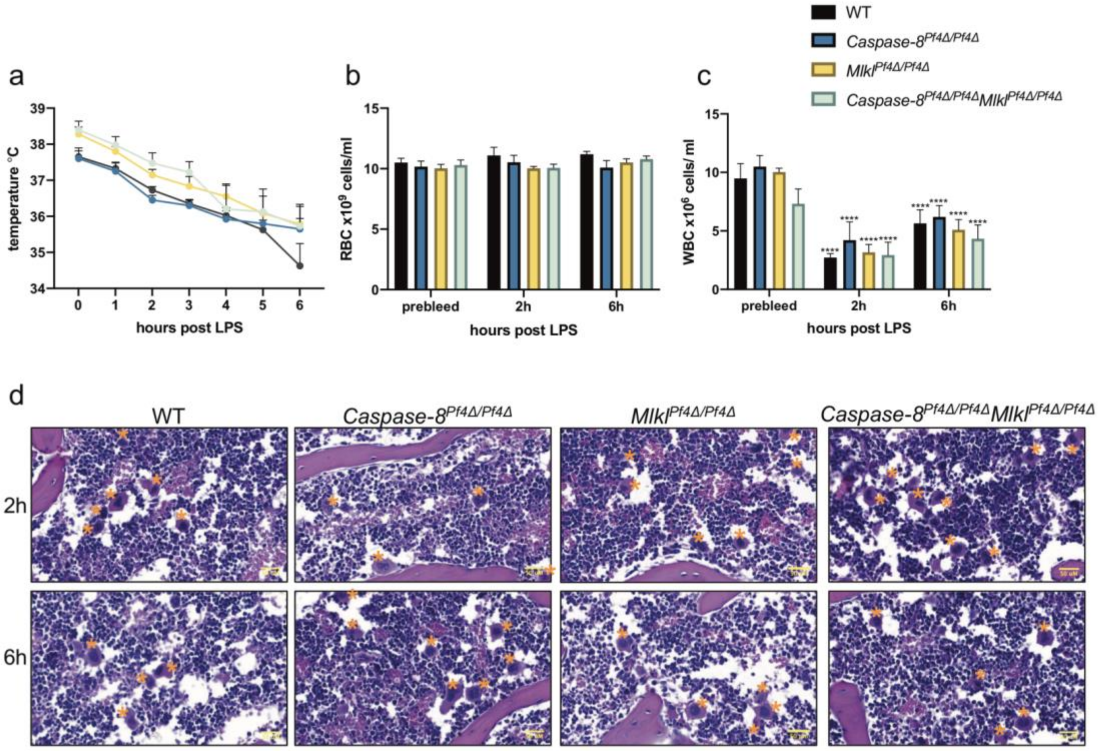

Supplementary figure 7: Responses of *Caspase-8*$^{Pf4\Delta/Pf4\Delta}$, *Mlkl*$^{Pf4\Delta/Pf4\Delta}$ and *Caspase-8*$^{Pf4\Delta/Pf4\Delta}$ *Mlkl*$^{Pf4\Delta/Pf4\Delta}$ mice post administration of LPS.

(a) Body temperature of WT, *Caspase-8*$^{Pf4\Delta/Pf4\Delta}$, *Mlkl*$^{Pf4\Delta/Pf4\Delta}$ and *Caspase-8*$^{Pf4\Delta/Pf4\Delta}$ *Mlkl*$^{Pf4\Delta/Pf4\Delta}$ mice administered with LPS over 6 hours, WT n=4, *Caspase-8*$^{Pf4\Delta/Pf4\Delta}$ n=4, *Mlkl*$^{Pf4\Delta/Pf4\Delta}$ n=6 and *Caspase-8*$^{Pf4\Delta/Pf4\Delta}$ *Mlkl*$^{Pf4\Delta/Pf4\Delta}$ n=4. **(b, c)** RBC and WBC counts at 2h and 6h post single I.P administration of LPS at 5mg/ml. Mice were analyzed in cohorts and blood was collected by cardiac puncture. 2h LPS; Floxed control (WT) n=3, *Caspase-8*$^{Pf4\Delta/Pf4\Delta}$ n=4, *Mlkl*$^{Pf4\Delta/Pf4\Delta}$ n=4 and *Caspase-8*$^{Pf4\Delta/Pf4\Delta}$ *Mlkl*$^{Pf4\Delta/Pf4\Delta}$ n=3. 6h LPS; WT n=4, *Caspase-8*$^{Pf4\Delta/Pf4\Delta}$ n=4, *Mlkl*$^{Pf4\Delta/Pf4\Delta}$ n=6, and *Caspase-8*$^{Pf4\Delta/Pf4\Delta}$ *Mlkl*$^{Pf4\Delta/Pf4\Delta}$ n=4. **(d)** Representative images of H&E stained sternal sections of mice with the indicated genotypes 2 and 6 hours post administration of LPS, megakaryocytes are indicated by asterisks (orange). Data represent mean ± s.d. Two-way ANOVA relative to untreated for WT, *Caspase-8*$^{Pf4\Delta/Pf4\Delta}$, *Mlkl*$^{Pf4\Delta/Pf4\Delta}$ and *Caspase-8*$^{Pf4\Delta/Pf4\Delta}$ *Mlkl*$^{Pf4\Delta/Pf4\Delta}$ with the Bonferroni multiple comparison test, ****$P<0.0001$.

Supplementary Table 1: Effect size and power calculations

Experiment	Effect size (Cohen's *d*)	Power (α=0.05)	Genotype	Mean	SD	N
**Figure 1 a**			$Mlkl^{WT}/Mlkl^{WT}$	2.35	0.45	5
$Mlkl^{WT}/Mlkl^{WT}$ vs $Mlkl/Mlkl^{D139V}$	0.98758425	0.2082207	$Mlkl^{WT}/Mlkl^{WT}$ vs $Mlkl/Mlkl^{D139V}$	1.81	0.62	3
$Mlkl^{WT}/Mlkl^{WT}$ vs $Mlkl^{D139V}/Mlkl^{D139V}$	3.85398586	0.9999537	$Mlkl^{D139V}/Mlkl^{D139V}$	0.85	0.32	7
**Figure 6 a**			WT	10.61	21.31	17
WT vs $Mlkl^{-/-}$	1.32321501	0.9559296	$Mlkl^{-/-}$	58.67	46.73	15
**Figure 7 d (2h LPS)**			WT	428.00	85.29	3
WT vs *Caspase-8* $^{PF4\Delta/PF4\Delta}$	7.53465189	1	*Caspase-8* $^{PF4\Delta/PF4\Delta}$	934.75	42.10	4
WT vs $Mlkl^{PF4\Delta/PF4\Delta}$	1.50103774	0.3578647	$Mlkl^{PF4\Delta/PF4\Delta}$	624.25	164.05	4
WT vs *Caspase-8* $^{PF4\Delta/PF4\Delta}$ $Mlkl^{PF4\Delta/PF4\Delta}$	5.3771831	0.9970253	*Caspase-8* $^{PF4\Delta/PF4\Delta}$ $Mlkl^{PF4\Delta/PF4\Delta}$	945.33	106.01	3
**Figure 7 d (6h LPS)**			WT	809.25	70.00	4
WT vs *Caspase-8* $^{PF4\Delta/PF4\Delta}$	2.92125559	0.9274194	*Caspase-8* $^{PF4\Delta/PF4\Delta}$	495.75	134.66	4
WT vs $Mlkl^{PF4\Delta/PF4\Delta}$	2.60702048	0.9407592	Mlkl PF4Δ/PF4Δ	650.17	50.47	6
WT vs *Caspase-8* $^{PF4\Delta/PF4\Delta}$ $Mlkl^{PF4\Delta/PF4\Delta}$	3.35913076	0.9744337	*Caspase-8* $^{PF4\Delta/PF4\Delta}$ $Mlkl^{PF4\Delta/PF4\Delta}$	609.00	46.98	4
**Supplementary figure 7 c (2 h LPS)**			WT	2.71	0.34	3
WT vs *Caspase-8* $^{PF4\Delta/PF4\Delta}$	1.32863076	0.2935264	*Caspase-8* $^{PF4\Delta/PF4\Delta}$	4.21	1.56	4
WT vs $Mlkl^{PF4\Delta/PF4\Delta}$	0.8658464	0.1531856	$Mlkl^{PF4\Delta/PF4\Delta}$	3.17	0.67	4
WT vs *Caspase-8* $^{PF4\Delta/PF4\Delta}$ $Mlkl^{PF4\Delta/PF4\Delta}$	0.27022867	0.0598033	*Caspase-8* $^{PF4\Delta/PF4\Delta}$ $Mlkl^{PF4\Delta/PF4\Delta}$	2.93	1.10	3
**Supplementary figure 7 c (6 h LPS)**			WT	5.64	1.1704415	4
WT vs *Caspase-8* $^{PF4\Delta/PF4\Delta}$	0.51829225	0.0954824	*Caspase-8* $^{PF4\Delta/PF4\Delta}$	6.1975	0.9717467	4
WT vs $Mlkl^{PF4\Delta/PF4\Delta}$	0.53031468	0.1123951	$Mlkl^{PF4\Delta/PF4\Delta}$	5.09	0.8839683	6
WT vs *Caspase-8* $^{PF4\Delta/PF4\Delta}$ $Mlkl^{PF4\Delta/PF4\Delta}$	1.11200445	0.2642289	*Caspase-8* $^{PF4\Delta/PF4\Delta}$ $Mlkl^{PF4\Delta/PF4\Delta}$	4.335	1.1767044	4
